# WadD, a New *Brucella* Lipopolysaccharide Core Glycosyltransferase Identified by Genomic Search and Phenotypic Characterization

**DOI:** 10.3389/fmicb.2018.02293

**Published:** 2018-09-27

**Authors:** Miriam Salvador-Bescós, Yolanda Gil-Ramírez, Amaia Zúñiga-Ripa, Estrella Martínez-Gómez, María J. de Miguel, Pilar M. Muñoz, Axel Cloeckaert, Michel S. Zygmunt, Ignacio Moriyón, Maite Iriarte, Raquel Conde-Álvarez

**Affiliations:** ^1^Instituto de Salud Tropical, Instituto de Investigación Sanitaria de Navarra, and Departamento de Microbiología y Parasitología, Universidad de Navarra, Pamplona, Spain; ^2^Unidad de Tecnología en Producción y Sanidad Animal, Centro de Investigación y Tecnología Agroalimentaria, Instituto Agroalimentario de Aragón – IA2 (CITA – Universidad de Zaragoza), Zaragoza, Spain; ^3^Institut National de la Recherche Agronomique, Université François Rabelais de Tours, UMR 1282, Nouzilly, France

**Keywords:** lipopolysaccharide (LPS), bacterial pathogenesis, vaccine development, virulence factor, glycosyltransferase, brucellosis, *Brucella*

## Abstract

Brucellosis, an infectious disease caused by *Brucella*, is one of the most extended bacterial zoonosis in the world and an important cause of economic losses and human suffering. The lipopolysaccharide (LPS) of *Brucella* plays a major role in virulence as it impairs normal recognition by the innate immune system and delays the immune response. The LPS core is a branched structure involved in resistance to complement and polycationic peptides, and mutants in glycosyltransferases required for the synthesis of the lateral branch not linked to the *O*-polysaccharide (O-PS) are attenuated and have been proposed as vaccine candidates. For this reason, the complete understanding of the genes involved in the synthesis of this LPS section is of particular interest. The chemical structure of the *Brucella* LPS core suggests that, in addition to the already identified WadB and WadC glycosyltransferases, others could be implicated in the synthesis of this lateral branch. To clarify this point, we identified and constructed mutants in 11 ORFs encoding putative glycosyltransferases in *B. abortus*. Four of these ORFs, regulated by the virulence regulator MucR (involved in LPS synthesis) or the BvrR/BvrS system (implicated in the synthesis of surface components), were not required for the synthesis of a complete LPS neither for virulence or interaction with polycationic peptides and/or complement. Among the other seven ORFs, six seemed not to be required for the synthesis of the core LPS since the corresponding mutants kept the *O*-PS and reacted as the wild type with polyclonal sera. Interestingly, mutant in ORF BAB1_0953 (renamed *wadD*) lost reactivity against antibodies that recognize the core section while kept the *O*-PS. This suggests that WadD is a new glycosyltransferase adding one or more sugars to the core lateral branch. WadD mutants were more sensitive than the parental strain to components of the innate immune system and played a role in chronic stages of infection. These results corroborate and extend previous work indicating that the *Brucella* LPS core is a branched structure that constitutes a steric impairment preventing the elements of the innate immune system to fight against *Brucella*.

## Introduction

Members of the genus *Brucella* are the etiologic agents of brucellosis, a worldwide spread zoonosis that affects ruminants, camelids, swine, dogs, and several forms of marine and terrestrial wildlife and causes abortions, infertility, and the subsequent economic losses in livestock. Humans become infected via direct contact with affected animals and through consumption of unpasteurized dairy products, and develop a chronic and debilitating condition that requires prolonged antibiotic treatment, being lethal in 1–5% of untreated cases (Ariza, 1999). Because of its impact on animal production and Public Health, it is estimated that brucellosis imposes a heavy burden in the developing world (McDermott et al., 2013).

The genus includes several nominal species that show host preferences^1^. Those that have been known for a long time (often referred to as “classical” *Brucella* species) include *B. abortus* and *B. melitensis* (the *brucellae* that infect domestic ruminants), *B. suis* (infecting swine, reindeer, hares, and several species of wild rodents), *B. canis* (infecting dogs), *B. ovis* (not zoonotic and restricted to sheep), and *B. neotomae* (infecting the desert woodrat). Because of their early identification and their economic and public health importance, *B. abortus, B. melitensis*, and *B. suis* are the best-characterized members of the genus, and all of them produce smooth (S) glossy colonies, a morphology that reflects the existence of a lipopolysaccharide (LPS) carrying an *O*-polysaccharide (O-PS) linked to the core-lipid A section that anchors the molecule to the outer membrane (OM). These *Brucella* spp. behave as facultative intracellular parasites of professional and non-professional phagocytes, an ability that depends on a number of virulence factors, chiefly a type IV secretion system and a peculiar OM structure. Critical OM components such as the S-LPS, lipoproteins, and ornithine lipids differ in relevant molecular details from the homologous molecules that in other bacteria bear the pathogen-associated molecular patterns (PAMP) readily detected by innate immunity pattern recognition receptors (PRRs). Consequently, these *brucellae* induce comparatively low and delayed proinflammatory responses, which create a time window allowing the pathogen to traffic intracellularly in dendritic cells and macrophages to reach a safe niche before effective phagocyte activation takes place (Lapaque et al., 2005; Barquero-Calvo et al., 2007; Palacios-Chaves et al., 2011). In this regard, the *Brucella* S-LPS carries the most significant PAMP modifications and is thus a major virulence factor (Lapaque et al., 2005). Whereas the structure of the *O*-PS (a *N*-formylperosamine homopolymer) and its role in virulence in animal models and in the natural host have been known for a long time, the importance of the core and its structure have only recently been established.

**FIGURE 1:**
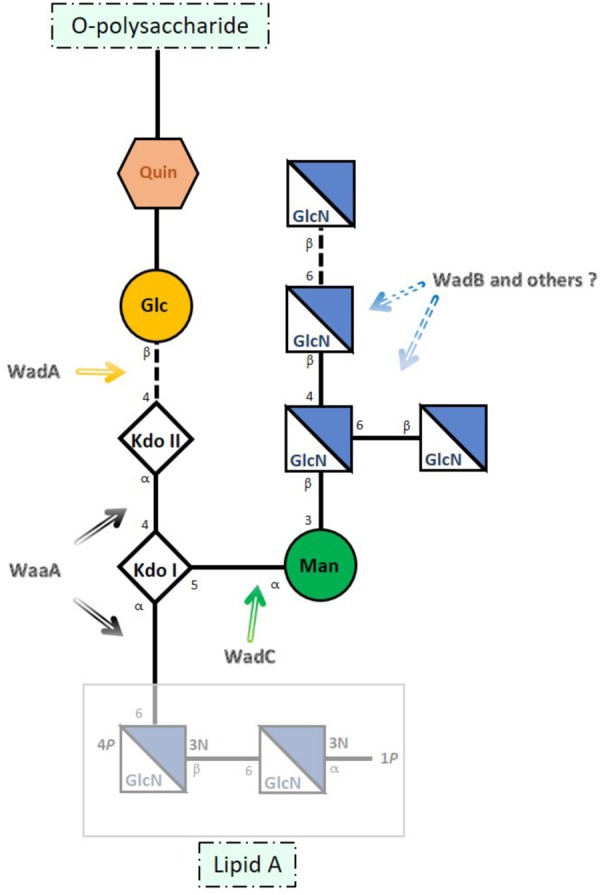
Schematic representation of *Brucella melitensis* LPS core. The site of action of the glycosyltransferases identified thus far is indicated with arrows. Kdo (3-deoxy-D-manno-octulosonic acid), Glc (glucose), Man (mannose), GlcN (glucosamine), and Quin (quinovosamine) [adapted from Fontana et al. (2016) and Gil-Ramírez et al. (2014)].

The core of *B. melitensis* LPS (**Figure 1**) is a branched oligosaccharide built of lipid A-linked 3-deoxy-D-manno-2-octulosonic acid (Kdo), glucose, 2-amino-2,6-dideoxy-D-glucose (quinovosamine), mannose, and 2-amino-2-deoxy-D-glucose [glucosamine (GlcN)] (Iriarte et al., 2004; Conde-Álvarez et al., 2012; Kubler-Kielb and Vinogradov, 2013; Gil-Ramírez et al., 2014; Fontana et al., 2016). This structure accounts for the *Brucella* LPS core overlapping epitopes (Rojas et al., 1994) an inner one comprising the Kdo residues plus the glucose bridging KdoII with the *O*-PS and an outer epitope encompassing the mannose and GlcN residues (Iriarte et al., 2004; González et al., 2008; Fontana et al., 2016). This last epitope plays a critical role in the binding of monoclonal antibodies (MoAbs) such as A68/24G12/A08 and A08/24D08/G09 (Conde-Álvarez et al., 2012; Fontana et al., 2016).

Accordingly, the reactivity with R-LPS-specific MoAbs strongly suggests that the structure elucidated for *B. melitensis* is conserved in the classical species (Bowden et al., 1995; Zygmunt et al., 2012). Moreover, availability of the corresponding structure of several mutants has also allowed assigning genes that upon mutation generate LPSs that lack [i.e., rough (R) LPS] or carry *O*-PS (**Figure 1**). Gene *wadA* corresponds to the enzyme linking KdoII and glucose, *wadC* to the mannosyltransferase acting on KdoI, and *wadB* to a glucosaminyltransferase involved in the assembly of the GlcN branch (González et al., 2008; Conde-Álvarez et al., 2012; Gil-Ramírez et al., 2014). These genes are highly conserved in the classical *Brucella* species (Monreal et al., 2003; Iriarte et al., 2004; González et al., 2008; Conde-Álvarez et al., 2012; Gil-Ramírez et al., 2014; Soler-Lloréns et al., 2016) and as expected, all *Brucella* genomes also carry a *waaA* homolog, the essential gene coding for the Kdo transferase of Gram-negative bacteria (Raetz and Whitfield, 2002; Iriarte et al., 2004). However, since most but not all glycosyltransferases involved in LPS synthesis are monofunctional (Raetz and Whitfield, 2002), it remains to be determined whether glucosaminyltransferases other than WadB are required for the synthesis of the GlcN branch.

Based on the complete structure of the core and the phenotype of mutants in *wadB* and *wadC*, it is postulated that the lack of acidic groups other than the two Kdo and lipid A phosphates and the mannose-GlcN branch account for the role of *Brucella* core in virulence both in cellular and animal models. By virtue of the density of amino groups and close position to the inner core and lipid A, the GlcN tetrasaccharide both neutralizes and sterically protects those inner anionic groups, thereby hampering binding of bactericidal peptides and PRRs such as the activators of the antibody-independent classical complement pathway and MD2, the TLR4 co-receptor. Accordingly, core defects bolster proinflammatory responses causing an activation of innate immunity earlier than that of the wild type, thereby generating attenuation (Conde-Álvarez et al., 2012; Gil-Ramírez et al., 2014; Soler-Lloréns et al., 2014; Fontana et al., 2016). Also, although both *wadB* and *wadC* mutants maintain an intact *O*-PS, attenuation in mice is more severe for the latter (Conde-Álvarez et al., 2012; Gil-Ramírez et al., 2014; Fontana et al., 2016) strongly suggesting a correlation between the extent of core damage and the intensity of the immunoactivation that brings about attenuation. A complete elucidation of the genetics of *Brucella* LPS core could confirm such a correlation and, since LPS core mutants represent a tool for developing a new generation of brucellosis vaccines (Conde-Álvarez et al., 2013; Zhao et al., 2017), also provide a graded array of possibilities. With these possibilities in mind, we investigated *B. abortus* 2308 genes annotated as glycosyltransferases for their possible involvement in LPS core synthesis and relevant biological effects.

## Materials and Methods

### Bacterial Strains and Growth Conditions

The bacterial strains and plasmids used in this study are listed in **Supplementary Table S1**. All bacteria were grown either on tryptic soy agar (TSA, Pronadisa) plates or in tryptic soy broth (TSB, Scharlau) or Mueller-Hinton broth (Becton Dickinson, Difco) at 37°C. Where indicated, growth media were supplemented with kanamycin (Km) at 50 mg/ml, nalidixic acid (Nal) at 25 mg/ml, ampicillin (Amp) at 100 mg/ml, and/or 5% sucrose. Bacterial growth rates were determined at 37°C in Mueller–Hinton broth (Becton Dickinson, Difco), using a Bioscreen C apparatus (Lab Systems). All strains were stored in skim milk at -80°C. Work with *Brucella* was performed at the Biosafety Level 3 (BSL-3) laboratory facilities of the “Centro de Investigación Médica Aplicada de la Universidad de Navarra” (CIMA) and “Centro de Investigación y Tecnología Agroalimentaria de Aragón” (CITA), Spain.

### DNA Manipulations and Analyses

Sequence data were obtained from *Kyoto Encyclopedia of Genes and Genomes* (KEGG^2^). Searches for DNA and protein homologies between *Brucella* species and other α-proteobacteria such as *Ochrobactrum, Rhizobium*, or *Agrobacterium* were carried out using KEGG, *Basic Local Alignment Sequence Tool* (BLAST^3^), and *Clustal Omega*^4^ from the *European Molecular Biology Laboratory – European Bioinformatics Institute* (EMBL-EBI^5^). New glycosyltransferase identification, using *B. abortus* 2308 was supported by *Carbohydrate-Active enZymes* database (CAZy^6^). Primers were designed using *Primer 3* input^7^ and synthesized by Sigma–Aldrich. Plasmid DNA was extracted with *Qiaprep spin Miniprep* (Qiagen GmbH). When needed, DNA was purified from agarose gels using *Qiack Gel* extraction kit (Qiagen) and sequenced by the *Servicio de Secuenciación* of CIMA.

### Construction of Mutants

Open-reading frames (ORFs) BAB2_0133, BAB2_0135, BAB2_0105, and BAB1_1620 were mutagenized by in frame non-polar deletion in *B. abortus* 2308W (**Supplementary Table S1**).

For the construction of *Ba*ΔBAB2_0133 mutant, we first generated two PCR fragments: oligonucleotides BAB2_0133**-**F1 (5′-GCGTTGGACAAGTTGAGGTT-3′) and BAB2_0133**-**R2 (5′-CATAGCGGTCGGTTAAATGC-3′) were used to amplify a 572 base pairs (bp) fragment including codons 1–38 of BAB2_0133, as well as 458 bp upstream of the BAB2_0133 start codon. Oligonucleotides BAB2_0133**-**F3 (5′-GTATCGCCAGCCAATTTACGTCCGTATTGGAAGCCAAGAA-3′) and BAB2_0133**-**R4 (5′-CAGTAACAAAAGGCCGCTAT-3′) were used to amplify a 442 bp fragment including codons 299–326 of BAB2_0133 and 355 bp downstream of the BAB2_0133 stop codon. Both fragments were ligated by overlapping PCR using oligonucleotides F1 and R4 for amplification, and the complementary regions between R2 and F3 for overlapping. The resulting fragment, containing the BAB2_0133 deletion allele, was cloned into pCR2.1 (Invitrogen), to generate plasmid pMSB-01, sequenced to ensure the maintenance of the reading frame, subsequently subcloned into the *Bam*HI and the *Xba*I sites of the suicide plasmid pJQK (Scupham and Triplett, 1997) and transformed into competent *E. coli* S17 λpir (Simon et al., 1983). The resulting suicide pJQK-derived plasmid was introduced into *B. abortus* 2308 by conjugation. The first recombination event (integration of the suicide vector in the chromosome) was selected by Nal and Km resistance, and the second recombination (excision of the mutator plasmid leading to construction of the mutant by allelic exchange) was selected by Nal and sucrose resistance and Km sensitivity. The resulting colonies were screened by PCR with primers F1 and R4 which amplified a fragment of 1014 bp in the mutant and 1794 bp in the sibling strain that keeps the wild-type gene. Primers BAB2_0133**-**F1 and BAB2_0133**-**R5 (5′-AAGACCCAGTAGTTAGCACT-3′) amplified a fragment of 919 bp only in the wild-type strain. The mutation generated results in the loss of the 80% of the ORF.

*Ba*ΔBAB2_0135 mutant was constructed following the same procedure and using oligonucleotides BAB2_0135**-**F1 (5′-TGGCGGCCGCTCTAGAACACCGGACTGCCTGATAA-3′) and BAB2_0135**-**R2 (5′-CGGGCAATTTCGGCATAG-3′) that amplified a 240 bp fragment including codons 1–40 of BAB2_0135, as well as 120 bp upstream of the BAB2_0135 start codon, and oligonucleotides BAB2_0135**-**F3 (5′-CTATGCCGAAATTGCCCGCCGGTTTGGAAATGCGGTCAA-3′) and BAB2_0135**-**R4 (5′-ATCCACTAGTTCTAGTTATGTAGCCGCCACCGTTT-3′) that amplified a 232 bp fragment including codons 441–478 of BAB2_0135 and 115 bp downstream of the BAB2_0135 stop codon. The resulting colonies were screened by PCR with primers F1 and R4 that amplified a fragment of 472 bp in the mutant and 1672 bp in the sibling strain that keeps the wild-type gene. Primers BAB2_0135**-**F1 and BAB2_0135**-**R5 (5′-CGATTGCCAGTCCCAGAAAG-3′) amplified a fragment of 628 bp only in the wild-type strain. The mutation generated results in the loss of the 84% of the ORF.

For the construction of *Ba*ΔBAB2_0105 mutant, oligonucleotides BAB2_0105**-**F1 (5′-GCGTGTTCTACAGCCATGAA-3′) and BAB2_0105**-**R2 (5′-CCGCCGAAATGTAGGAAGTG-3′) amplified a 198 bp fragment including codons 1–33 of BAB2_0105, as well as 99 bp upstream of the BAB2_0105 start codon. Oligonucleotides BAB2_0105**-**F3 (5′-CACTTCCTACATTTCGGCGGTATGTTGGATTGGGACGGGT-3′) and BAB2_0105**-**R4 (5′-GCCGAATATGACGCTTGCTA-3′) amplified a 154 bp fragment including codons 307–330 of BAB2_0105 and 79 bp downstream of the BAB2_0105 stop codon. The resulting colonies were screened by PCR with primers F1 and R4 which amplified a fragment of 352 bp in the mutant and 1171 bp in the sibling strain which keeps the wild-type gene. Primers BAB2_0105**-**F1 and BAB2_0105**-**R5 (5′-CAAAGACCGGATATTGCGGG-3′) amplified a fragment of 550 bp only in the wild-type strain. The mutation results in the loss of the 83% of the ORF.

*Ba*ΔBAB1_1620 mutant was constructed using oligonucleotides 1620-F1 (5′-GTACGCGGTCGTAGCTCAGT-3′) and 1620-R2 (5′-CTCAAACTGAGACGCCATGA-3′), that amplified a 475 bp fragment including codons 1–23 of BAB1_1620 as well as 406 bp upstream of the ORF start codon. Oligonucleotides 1620-F3 (5′-TCATGGCGTCTCAGTTTGAGATAGCCAACGTCACCAAAACA-3′) and 1620-R4 (5′-CTCTGCAATTCTTGCGATCA-3′) were used to amplify a 410 bp fragment including codons 241–261 of the BAB1_1620 ORF and 347 bp downstream of the BAB1_1620 stop codon. Both fragments were ligated, cloned into pCR2.1 to generate plasmid pYRI-16, and subcloned into the suicide pJQK (pYRI-17). After conjugation with *B. abortus*, the resulting colonies were screened by PCR with primers 1620-F1 and 1620-R4 which amplified a 885 bp fragment in the mutant and 1536 bp in the parental strain. The mutation generated results in the loss of the 83% of the BAB1_1620 ORF.

The rest of the ORFs were mutagenized by recombination and gene disruption using as suicide vectors pJQK or pSKoriT (Tibor et al., 2002) carrying an internal fragment of the ORF.

For the construction of *Ba*::pJQK-BAB1_0114 mutants, we generated a PCR fragment using oligonucleotides BAB1_0114-F1 (5′-TCAACAAATCGGCCAAGGAC-3′) and BAB1_0114-R2 (5′-GTCACGCGGTCAAACTGG-3′) which amplified a 481 bp fragment containing the region that codes for amino acids 248–407. The fragment was cloned into pCR2.1, to generate plasmid pMSB-17, sequenced and subcloned into the *Bam*HI and the *Xba*I sites of the suicide plasmid pJQK to obtain pMSB-28, and then transformed into competent *E. coli* S17 and transferred into *B. abortus* 2308 by conjugation. The integration of the suicide vector and disruption of the target gene were selected by Nal and Km resistance and by PCR combining BAB1_0114-F3 (5′-CCTATATTCCCCAGGCCGTT-3′) with M13 Forward (5′-CTGGCCGTCGTTTTAC-3′) or with M13 Reverse (5′-CAGGAAACAGCTATGAC-3′). These last two primers hybridize in the suicide vector inserted in the chromosome. BAB1_0114-F3 and M13 Forward amplified a fragment of 881 bp only in the mutant strain. Following the same strategy, we constructed the rest of insertion mutants:

Mutant *Ba*::pSKoriT-BAB1_0417 was obtained using oligonucleotides BAB1_0417-F1 (5′-TGATCGACCATGGCTCGG-3′) and BAB1_0417-R2 (5′-TCAAGCCTGACCAGAAGCC-3′) which amplified a 295 bp fragment of BAB1_0417 (codon 37–134). The fragment was first cloned in pCR2.1 (pMSB-05), subcloned into the suicide plasmid pSKoriT (pMSB-06), and transferred into *B. abortus* 2308 by conjugation. Primers M13 Reverse and BAB1_0417-F3 (5′-CTGTTTCCCGACCAGCTTG-3′) amplified a fragment of 649 bp only in the mutant.

Oligonucleotides BAB2_0693-F1 (5′-CACTGCAAGCCGGTTACAAT-3′) and BAB2_0693-R2 (5′-TGCAACGAAATTCTGTCCGG-3′) were used for the construction of *Ba*::pJQK-BAB2_0693 mutant. F1 and R2 amplified a fragment of 416 bp (codons 249–386). We generated plasmid pMSB-16, subsequently subcloned into the suicide plasmid pJQK (pMSB-24), and conjugated into *B. abortus* 2308. Primers M13 Forward and BAB2_0693-F3 (5′-ACGAGCGCTATGATTTCGTC-3′) amplified a fragment of 684 bp only in the mutant.

For the construction of *Ba*::pJQK-BAB1_0932 mutants we used oligonucleotides BAB1_0932-F1 (5′-GCCGTCGTCCTGAATGTTAC-3′) and BAB1_0932-R2 (5′-GCCATTATCCAGTGCAGCC-3′) which amplified a 420 bp fragment of BAB1_0932 (codons 354–493). We generated plasmid pMSB-28, subsequently subcloned into the suicide plasmid pJQK (pMSB-29), and conjugated into *B. abortus* 2308. The resulting Nal–Km-resistant colonies were screened by PCR. Primers M13 Reverse and BAB1_0932-F3 (5′-GGCCGAGAATGGCTATATCA-3′) amplified a fragment of 915 bp only in the mutant.

Mutant *Ba*::pSKoriT-BAB1_0326 was obtained using oligonucleotides BAB1_0326-F1 (5′-GCACTCAACCGGCTCAATTG-3′) and BAB1_0326-R2 (5′-AGCACCGCATATTCAAAGGC-3′) which amplified a 368 bp fragment of BAB1_0326 (codons 261–383) that was cloned into pCR2.1 to obtain pMSB-07. The fragment was then subcloned into the suicide pSKoriT (pMSB-10), and conjugated into *B. abortus* 2308. The resulting Nal–Km-resistant colonies were screened by PCR. Primers M13 Reverse and BAB1_0326-F3 (5′-ATGTTGCCATGTCGCTGTTT-3′) amplified a fragment of 678 bp only in the mutant strain.

Construction of *Ba*::pJQK-BAB1_0607 mutants was carried out using oligonucleotides BAB1_0607-F1 (5′-GCCAATGTCGTTCTCTCCAA-3′) and BAB1_0607-R2 (5′-CTTGGTGTCAGCCCCTTTTC-3′) which amplified a 449 bp fragment of BAB1_0607 (codons 278–427). We generated the pCR2.1-derived plasmid pMSB-19, then subcloned into the suicide plasmid pJQK (pMSB-21), and conjugated into *B. abortus* 2308. Primers M13 Forward and BAB1_0607-F3 (5′-TTCTTTCCAATGAGCGCACC-3′) amplified a fragment of 800 bp only in the mutant.

We constructed two different mutants in ORF BAB1_0953 (*wadD*). The first, *Ba*::pSKoriT-BAB1_0953, carried the suicide vector inserted in the gene and was obtained with oligonucleotides BAB1_0953-F1 (5′-ACTTTTCGCCGAGCAACAAA-3′) and BAB1_0953-R2 (5′-AGGCACGGTTTCATAGACGA-3′) which amplified a 358 bp fragment of BAB1_0953 (codons 112–230). We generated plasmid pMSB-11, subsequently subcloned into the suicide plasmid pSKoriT (pMSB-12), and conjugated into *B. abortus* 2308. Primers M13 Forward and BAB1_0953-F3 (5′-GCTGGCTTCATGAAATCCGT-3′) amplified a fragment of 612 bp in mutant.

We also constructed a non-polar *wadD* mutant (*BaΔwadD)* by in frame deletion. Oligonucleotides *wadD*-F1 (5′-TCTATAATGAGAGGCGGCTTTT-3′) and *wadD*-R2 (5′-AGAAGTGCTGGTCCTGTTGT-3′) were used to amplify a 304 (bp) fragment including codons 1–50 of BAB1_0953, as well as 154 bp upstream of the BAB1_0953 start codon. Oligonucleotides *wadD*-F3 (5′-ACAACAGGACCAGCACTTCTATCCTCACCCTGCCATTCAA-3′) and *wadD*-R4 (5′-CTGGTACTAGACGCCCTGTT-3′) were used to amplify a 175 bp fragment including codons 281–324 of BAB1_0953 and 43 bp downstream of the BAB1_0953 stop codon. Both fragments were ligated by overlapping PCR using oligonucleotides F1 and R4 for amplification, and the complementary regions between R2 and F3 for overlapping. The resulting fragment, containing the BAB1_0953 deletion allele, was cloned directly into pJQK by the *InFusion* cloning system (Clontech) to generate pMSB-34. This suicide vector was sequenced to ensure the maintenance of the reading frame and transferred into *B. abortus* 2308 by conjugation. The resulting colonies were screened by PCR with primers F1 and R4 that amplified a fragment of 479 bp in the mutant and 1169 bp in the sibling strain which keeps the wild-type gene. Primers *wadD*-F1 and *wadD*-R5 (5′-AGGCACGGTTTCATAGACGA-3′) amplified a fragment of 844 bp only in the wild-type strain. The mutation generated results in the loss of the 71% of the ORF and the mutant was called *BaΔwadD*.

### Complementation of *wadD* Mutants

For complementation experiments, we performed a stable insertion of the miniTn7 transposon into the chromosome of *BaΔwadD* (Choi and Schweizer, 2006). For this purpose, we first generated a PCR product using oligonucleotides Tn7-*wadD*-F1 (5′-CGGGCTGCAGGAATTGCGATTCCTTTGTGCCAGAT-3′) and Tn7-*wadD*-R2 (5′-GCTTCTCGAGGAATTATCATCGCCGCATTGAAGAC-3′), which amplified a 1771 bp fragment including codons 1–323 of BAB1_0953 together with 481 bp upstream of the ORF start codon including the putative *wadD* promoter and 318 bp downstream the ORF stop codon. This PCR product was cloned into the corresponding sites of the linearized pUC18 R6KT miniTn7T Km^R^ vector (Llobet et al., 2009) to generate plasmid pMSB-44. The plasmid was sequenced to ensure the maintenance of the reading frame transformed into *E. coli* S17 and transferred to *BaΔwadD* mutant by tetra-parental conjugation between *E. coli* S17 (pMSB44), *E. coli* SM10 λpir (pTNS2), and *E. coli* HB101 (pRK2013). The conjugants harboring pMSB-44 were selected by plating the mating mixture onto TSA–Nal–Km plates that were incubated at 37°C for 4 days. To confirm that the transposon was inserted between genes *glmS* and *recG* (Choi and Schweizer, 2006), we performed PCR using different oligonucleotides: Tn7F (5′-TGGCTAAAGCAAACTCTTCATTT-3′) and Tn7R (5′-GCGGATTTGTCCTACTCAGG-3′) allowed to confirm that the Tn7 was inserted, oligonucleotides Glms_B (5′-GTCCTTATGGGAACGGACGT-3′) and PTn7-R (5′-CACAGCATAACTGGACTGATT-3′) confirmed that the transposon was inserted immediately after the gene *glmS*, and RecG-R (5′-TATATTCTGGCGAGCGATCC-3′) and PTn7-L (5′-ATTAGCTTACGACGCTACACCC-3′) confirmed that the transposon was inserted before the gene *recG* (Choi and Schweizer, 2006). The resulting strain was named *BaΔwadD*::Tn7-P*wadD*.

### Crystal Violet Exclusion Test

To study if the mutants had smooth (complete LPS) or rough (*O*-PS-lacking LPS) phenotype, 5 ml of a crystal violet solution at 0.1 mg/ml in distilled water were used to cover isolated colonies on TSA plates for 20 s. Smooth colonies excluded crystal violet and looked white, whereas rough colonies captured crystal violet and looked violet.

### LPS Extraction and Characterization

LPS was extracted by the proteinase-K sodium dodecyl sulfate (SDS) protocol (Dubray and Limet, 1987; Garin-Bastuji et al., 1990) with some modifications. Bacteria grown overnight in 10 ml of TSB were killed with 0.5% phenol during 3 days in agitation at 37°C. After that, samples were weighed and pipetted into small polycarbonate cap tubes and then suspended by ultrasounds in 2% SDS–60 mM Tris–HCl buffer (pH 6.8) at a concentration of 0.5 g (wet weight) of bacteria per 10 ml of buffer. Samples were then heated at 100°C for 10 min, and lysates were cooled to 55°C. This treatment was followed by digestion with 60 μl of proteinase-K at 2.5 mg/ml in HCl–Tris per ml of sample (Merck KGaA) for 3 h at 55°C, and overnight incubation at 20°C. Afterward, they were centrifuged at 20,000 × *g* for 30 min at room temperature, and the LPS was precipitated from the supernatant by addition of 3 volumes of methanol containing 1% sodium acetate-saturated methanol at -20°C. After 60 min, the precipitate was harvested by centrifugation at 5,000 × *g* for 15 min at 4°C and resuspended by sonication in 10 ml of distilled water. After a second methanol precipitation and centrifugation, the pellets were resuspended by sonication in 2–3 ml of 60 mM HCl–Tris (pH 6.8) and left at 37°C. Then samples were treated with 20 μl/ml of RNase and DNase stock solutions at 0.5 mg/ml in HCl–Tris (MP Biomedicals and Sigma–Aldrich, respectively) at 37°C for 30 min. Subsequently, the LPS was treated again with 5 μl/ml of proteinase K at 2.5 mg/ml in HCl–Tris, at 55°C for 3 h and then, at room temperature overnight. After a third methanol precipitation in the same conditions described above, the pellet containing LPS was recovered in 1 ml of distilled water and frozen at -20°C.

### SDS–PAGE and Western Blots

Samples were mixed 1:1 with Sample buffer 2× (Bio-Rad), heated at 100°C for 10 min, and analyzed in Tris–HCl–glycine-12, 15, or 18% polyacrylamide gels (37.5:1 acrylamide/methylene–bisacrylamide ratio). Fifteen microliters of each sample were run at 30 mA constant current for 140 min. Finally, LPS molecules were revealed by the periodate-alkaline silver method (Tsai and Frasch, 1982).

For Western blot, gels were electro-transferred onto PVDF sheets (Whatman, Schleicher & Schuell, WESTRAN S.; 0.2 μm pore size) in a transfer buffer (pH 8.3) containing 0.025 M Tris, 0.192 M glycine, and 20% (vol/vol) methanol. Transfer was performed at a constant voltage of 8 V and 200 mA for 30 min in a Trans-Blot Semi-Dry Transfer Cell (Bio-Rad). Antibodies used were MoAbs A68/24D08/G09 and A68/24G12/A08, which recognize core epitopes (Bowden et al., 1995), and a polyclonal serum from a rabbit infected with *B. melitensis* 16 M and bled at day 45.

### Enzyme-Linked Immunosorbent Assay (ELISA)

Enzyme-Linked Immunosorbent Assay using whole bacteria (sonicated cells) as the antigen were performed as described previously (Cloeckaert et al., 1993a). MoAbs used were directed against *O*-PS, R-LPS, and the OM lipoproteins Omp10, Omp16, and Omp19 (Cloeckaert et al., 1990, 1993a,b, 1998). The anti-R-LPS MoAbs used were A68/03F03/D05 (IgG2b), A68/10A06/B11 (IgM), A68/24D08/G09 (IgG1), and A68/24G12/A08 (IgG3). The MoAbs specific for the *O*-PS epitopes were 2E11 (IgG3; M epitope), 12G12 [IgG1; C (A = M) epitope], 07F09 [IgG1; C (A = M) epitope], 12B12 [IgG3; C (M > A) epitope], 18H08 [IgA; C/Y (A = M) epitope], 04F9 [IgG2a; C/Y (A > M) epitope], and 05D4 [IgG1; C/Y (A > M) epitope]. The MoAbs specific for OM lipoproteins were A68/08E07/B11 (Omp10; IgG2a), A68/04G01/C06 (Omp16; IgG2a), A76/08C03/G03 (Omp16; IgG2a), and A76/10D03/H02 (Omp19; IgG2b). All MoAbs were used as hybridoma supernatants in ELISA.

### Sensitivity to Polycationic Bactericidal Peptides

The minimal inhibitory concentration (MIC) of polymyxin B and poly-L-ornithine (both from Sigma–Aldrich) were determined in Mueller–Hinton medium. Exponentially growing bacteria were adjusted to an OD equivalent to 1 of the McFarland scale, and exposed to serial dilutions of the bactericidal peptides. MICs were determined by technical duplicates after 2 days of incubation at 37°C. Experiments were performed in triplicate.

### Sensitivity to the Bactericidal Action of Non-immune Serum

Exponentially growing bacteria were adjusted to 10^4^ Colony Forming Units (CFU)/ml in saline and dispensed in duplicate in microtiter plates (30 μl/well) containing 60 μl of new-born bovine serum. After 90 min of incubation at 37°C with gentle agitation, complement action was blocked by adding brain heart infusion (BHI) broth (150 μl/well). After mixing the BHI broth with the bacterial suspension, 75 μl were plated by triplicate on TSA plates. Five days after incubation at 37°C, results were expressed as the percentage of CFU recovered with respect to control samples where new-born bovine serum was substituted by PBS. The experiment was repeated three different times.

### Virulence in Mice

Seven-week-old female BALB/c mice (ENVIGO, Harlan) were lodged in cages in BSL-3 facilities with water and food *ad libitum* for 2, 8, or 12 weeks. Six groups of five mice each were inoculated with *BaΔwadD* or *Ba*-parental. Inocula were prepared in sterile PBS and each mouse was administered intraperitoneally approximately with 5 × 10^4^ CFU in 0.1 ml. To assess the exact dose retrospectively, dilutions of each inoculum were plated by triplicate on TSA plates. Spleen CFUs in infected mice were counted at 2, 8, and 12 weeks after inoculation. The CFU counts were normalized by logarithmic transformation and the mean log CFU/spleen values and the standard deviations were calculated. The spleens were weighed and homogenized in 9 volumes of PBS and serial 10-fold dilutions were accomplished and plated by triplicate on TSA plates. After 5 days of incubation at 37°C the colonies were checked by crystal violet exclusion test and PCR.

### Statistical Analysis

Statistical significance for sensitivity to normal serum was evaluated with one-way ANOVA followed by Dunnett’s multiple comparisons test (^∗∗∗∗^*p* < 0.0001). For virulence analysis, statistical significance between the parental strain and the *wadD* mutant was evaluated using Student’s *t*-independent-samples test (^∗^*p* < 0.05).

## Results

### Screening for Putative LPS Core Glycosyltransferases

A bioinformatic search in the Carbohydrate-Active Enzymes database CAZy^8^ revealed 23 ORFs in the genome of *B. abortus* 2308 (**Supplementary Table S1**) that could code for glycosyltransferases. We excluded from further analysis BAB1_0108-*cgs*, which is involved in cyclic glucan synthesis (Briones et al., 2001), BAB1_1786-*mtgA* and BAB1_1450-*murG*, both related to peptidoglycan synthesis, BAB1_1171-*lpxB*, probably implicated in lipid A formation (Iriarte et al., 2004), and BAB1_0553-*wbkA*, BAB1_0563-*wbkE*; BAB1_1000-*wboA* and BAB1_1000-*wboB*, four genes that belong to the *O*-PS synthesis route (McQuiston et al., 1999; Godfroid et al., 2000; González et al., 2008). Similarly, four ORFs correspond to those glycosyltransferases already known to be involved in the synthesis of the LPS core: BAB1_0639-*wadA* (Monreal et al., 2003), BAB1_0351-*wadB* (Gil-Ramírez et al., 2014), BAB1_1522-*wadC* (Conde-Álvarez et al., 2012), and BAB2_0209-*waaA* (Iriarte et al., 2004) (see the section “Introduction”). The remaining 11 ORFs are listed in **Table 1**, and data on their presence in other *Brucella* spp. and genetic location are in the Supplementary Material (**Supplementary Table S2** and **Supplementary Figure S1**). Of these, seven (BAB1_0953, BAB2_0105, BAB2_0133, BAB2_0135, BAB1_1620, BAB1_0607, and BAB1_0932) were highly conserved in all *Brucella* spp., but four (BAB2_0693, BAB1_0417, BAB1_0114, and BAB1_0326) presented significant differences when compared to *B. abortus* sequences, mainly due to frameshifts generating shorter proteins (**Supplementary Table S2**). Perusal of the literature revealed some information on 4 of those 11 putative glycosyltransferases. Expression of BAB1_0326, BAB2_0133, and BAB2_0135 has been shown to be controlled by MucR, a general virulence regulator in *Brucella* (Caswell et al., 2013). However, although it has been reported that *B. abortus* and *B. melitensis mucR* mutants have a defective LPS core, the glycosyltransferases involved have not been identified (Caswell et al., 2013; Mirabella et al., 2013). Also, BAB1_1620 expression has been reported to be controlled by BvrR/BvrS, a master regulator of *Brucella* virulence that modulates OM homeostasis and undetermined aspects of LPS structure (Manterola et al., 2005; Viadas et al., 2010).

**Table 1:** ORF coding for *B. abortus* hypothetical glycosyltransferases, family to which they belong, predicted function, and the corresponding mutant LPS phenotype by Western blot analysis.

ORF	Family	Predicted function (KEGG)	Mutant LPS reactivity	
			*O*-PS^a^	R-LPS^b^
BAB1_0326	2	Glycosyltransferase	+	+
BAB2_0133	2	Glycosyltransferase	+	**+**
BAB2_0105	2	Glycosyltransferase	+	+
BAB2_0693	2	Glycosyltransferase	+	**+**
BAB1_0953	2	Glycosyltransferase	+	**-**
BAB1_1620	25	Glycosyltransferase	+	+
BAB1_0607	51	Penicillin-binding protein 1A transpeptidase domain – Glycosyltransferase	+	+
BAB1_0114	51	Penicillin-binding protein transpeptidase domain: ATP/GTP-binding site motif A (P-loop) – Glycosyltransferase	+	+
BAB1_0932	51	Penicillin-binding protein 1A transpeptidase domain – Glycosyltransferase	+	+
BAB2_0135	83	Possible dolichyl-phosphate-mannose-protein mannosyltransferase family protein	+	+
BAB1_0417	nc^c^	Conserved hypothetical protein	+	+

*^*a*^Reactivity to polyclonal serum from a rabbit infected with *B. melitensis* 16M. ^*b*^Reactivity to monoclonal antibodies anti-R-LPS: A68/24G12/A08 and A68/24D08/G09. ^*c*^Glycosyltransferase family non-classified.*

We first analyzed the MucR and BvrR/BvrS controlled ORFs for involvement in LPS synthesis. To this end, we constructed an insertion mutant in BAB1_0326 (since the downstream ORF is oriented in the opposite direction) as well as non-polar deletion mutants in BAB2_0133 and BAB2_0135 (both part of an operon), and BAB1_1620 (which, although isolated, is surrounded by genes implicated in the cell cycle). These four mutants maintained the S phenotype in the crystal violet assay, suggesting that they kept an intact *O*-PS. Then, SDS–proteinase-K LPSs were analyzed by SDS–PAGE and Western blot with both a polyclonal serum against S *brucellae* and anti-core MoAbs A68/24G12/A08 and A68/24D08/G09, using as controls LPS from *B. abortus* 2308W (*Ba*-parental), a *B. abortus wadC* mutant (*BaΔwadC*), and a R *per* mutant (*BaΔper*) (Martínez-Gómez et al., 2018). The LPS of the four mutants presented S and R fractions with migration profiles identical to those of *Ba*-parental LPS and reacted similarly with the serum and MoAbs (**Supplementary Figure S2**) strongly suggesting that the corresponding ORFs are not required for normal LPS synthesis. When we complemented these observations by inoculating BALB/c mice with BAB2_0133, BAB2_0135, and BAB1_1620 mutants, they produced CFU/spleen that did not differ from those of *Ba*-parental at weeks 2 (*p* = 0.99; 0.75 and 0.45, respectively) and 8 (*p* = 0.95; 0.99 and 0.99) after infection. Moreover, mutants in BAB2_0133 and BAB1_1620 behaved similarly to *Ba*-parental in polycationic peptide resistance, and the former also performed as *Ba*-parental in sensitivity to normal serum (**Supplementary Figure S3**). These results are consistent with the idea that the putative glycosyltransferases regulated by MucR or BvrR/BvrS are not involved in the synthesis of LPS or of other components implicated in virulence, at least under the conditions used in this study.

**FIGURE 2:**
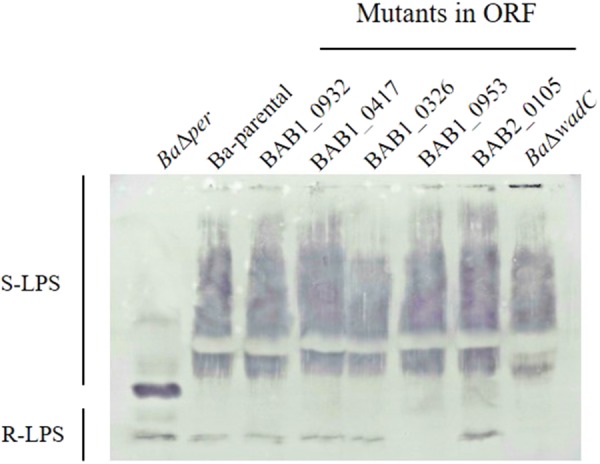
Mutation of *B. abortus* BAB1_0953 but not of other candidate for LPS glycosyltransferase genes abrogates reactivity of the R-LPS but not of the S-LPS fraction with antibodies from a *B. melitensis*-infected rabbit. *Ba*Δ*per* is a *N*-formyl-perosamine synthase mutant lacking the LPS *O*-PS, and *Ba*Δ*wadC* is a LPS mutant bearing *O*-PS but defective in the mannose-tetraglucosamine lateral branch of the core oligosaccharide (**Figure 1**).

To investigate whether the remaining seven putative glycosyltransferases (BAB1_0953, BAB2_0105, BAB2_0693, BAB1_0607, BAB1_0114, BAB1_0932, and BAB1_0417) were required for LPS synthesis, we constructed *B. abortus* 2308W insertion mutants in each of them. All mutants were S by the crystal violet assay and the analysis of the extracted LPS showed S fractions with a migration profile like that of *Ba*-parental and reacted similarly with the anti S-*Brucella* polyclonal serum (**Figure 2** and **Supplementary Figure S4**). Interestingly, although keeping the S fraction, mutant in BAB1_0953 lost reactivity in the R fraction, suggesting a defect in the core and/or lipid A epitope(s) recognized by polyclonal sera of infected animals (Rojas et al., 2001). Since this was not observed for the other mutants, we investigated further BAB1_0953 and the phenotype associated with its mutation.

### BAB1_0953 Encodes WadD, a Previously Unidentified Glycosyltransferase Involved in the Synthesis of the LPS Core Lateral Branch

**FIGURE 3:**
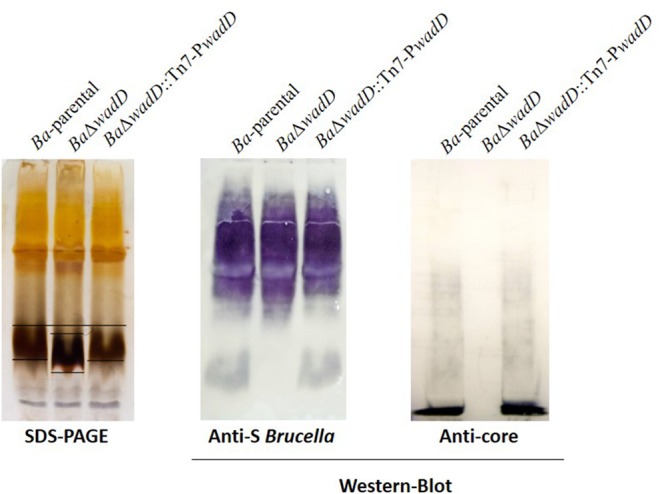
Mutation of *wadD* generates an LPS core defect. Left panel, SDS–PAGE electrophoresis and silver staining of SDS–proteinase K extracts; central panel, Western blot analysis of SDS–proteinase K extracts with a polyclonal serum of a *B. melitensis*-infected rabbit; right panel, Western blot analysis of SDS–proteinase K extracts with monoclonal anti-core antibody A68/24G12/A08.

**FIGURE 4:**
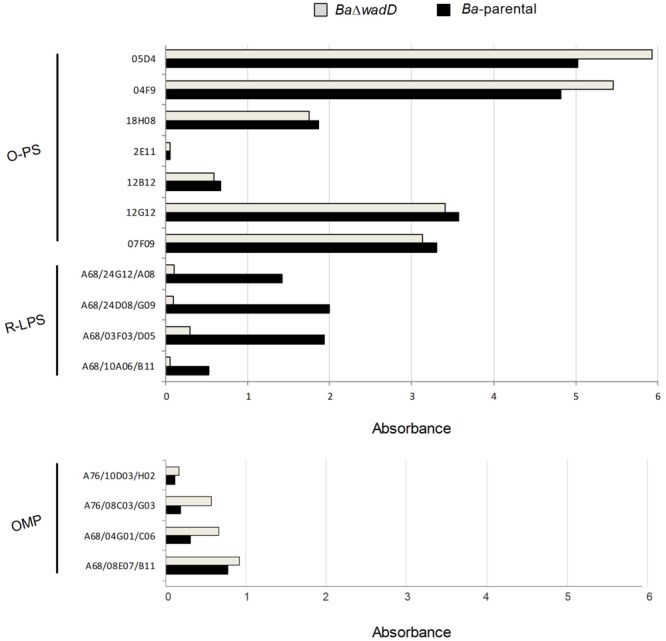
Outer membrane epitopes in *Ba*Δ*wadD* and *Ba*-parental. Upper panel, anti-*O*-PS and anti-R-LPS; lower panel, anti-OMP antibodies measured by ELISA test.

BAB1_0953 is an isolated gene and the adjacent ORFs are encoded in the complementary strand. Thus, it was very unlikely that a polar effect caused the LPS phenotype of the insertion mutant. However, to rule out such a possibility, we constructed a non-polar deletion mutant, hereafter named *Ba*Δ*wadD* following the nomenclature previously established for *Brucella* LPS core genes (Reeves et al., 1996; Gil-Ramírez et al., 2014). *Ba*Δ*wadD* LPS showed a migration profile similar to that of *Ba*-parental in the high molecular weight S-LPS fraction and an increased mobility in the R-LPS one. Western-blot analysis with a polyclonal serum showed that, while the former fraction kept the reactivity with this serum, the latter failed to react indicating a significant alteration of the core-lipid A epitopes. To assign the defect to the core oligosaccharide, we probed the LPS with MoAbs A68/24G12/A08 and A68/24D08/G09, the binding of which to the R-LPS requires an intact mannose-GlcN tetrasaccharide (Conde-Álvarez et al., 2012; Fontana et al., 2016). Both antibodies failed to react with the R-LPS fraction and this failure was reverted upon insertion of a complete *wadD* gene in the bacterial chromosome of the deletion mutant (*Ba*Δ*wadD*::Tn7-P*wadD*). Moreover, this complementation restored both the migration pattern of the R-LPS fraction to the level of the *Ba*-parental LPS and the reactivity with the polyclonal serum (**Figure 3**). An ELISA with several anti-core MoAbs and whole bacteria confirmed the core defect (**Figure 4**, upper panel).

We only observed small but constant differences in reactivity of *Ba*-parental and *Ba*Δ*wadD* with anti-Outer Membrane Proteins (OMP) antibodies (**Figure 4**, lower panel). Although considering the limitations of the method used, this suggests that the presence of the *O*-PS and the defect in the core LPS could generate a steric hindrance that would allow the access of antibodies to the OMPs (Bowden et al., 1995) or the possibility that *wadD* mutation could affect the amount of LPS in the OM or how it is inserted in the bacterial surface.

Although as signaled above, the final confirmation would require a complete chemical analysis, all these results strongly suggest that *wadD* encodes a previously unidentified glycosyltransferase involved in the synthesis of the core lateral branch.

### WadD Orthologs Are Present in all *Brucella* spp. but in a Recently Characterized Isolate From Amphibians

*In silico* analysis (**Supplementary Figure S5**) showed that *wadD* was highly conserved in the core *brucellae* including the “classical” spp. *B. melitensis, B. suis* (smooth LPS), and *B. ovis* and *B. canis* (rough LPS) and also in other “non-classical” smooth *Brucella* spp.: *B. pinnipedialis, B. microti, B. ceti*, and *B. vulpis*. We also analyzed the presence of *wadD* in the group of early-diverging *brucellae* that depart from the classical spp. and includes *B*. *inopinata* strain BO1, *B. inopinata*-like strain BO2, an isolated strain from native rodents in Australia (NF2653), and the *Brucella* spp. recently isolated from amphibians. These early-diverging *Brucella* produce an atypical LPS (Scholz et al., 2010; Tiller et al., 2010; Soler-Lloréns et al., 2016; Al Dahouk et al., 2017). Unexpectedly, *wadD* was present in all of them but absent in *Brucella* spp. B13-0095, one of the four *Brucella* strains isolated from frogs that have been completely sequenced (Soler-Lloréns et al., 2016). In contrast, this strain conserves *wadB* and *wadC*. Finally, WadD was 72 and 71% homologous to *Ochrobactrum anthropi* and *O. intermedium* orthologs, respectively, two species that also belong to the α-2 *Proteobacteria* subclass and are the closest genetic neighbors of *Brucella*.

### Dysfunction of *wadD* Generates Increased Sensitivity to Cationic Peptides and Normal Serum

**FIGURE 5:**
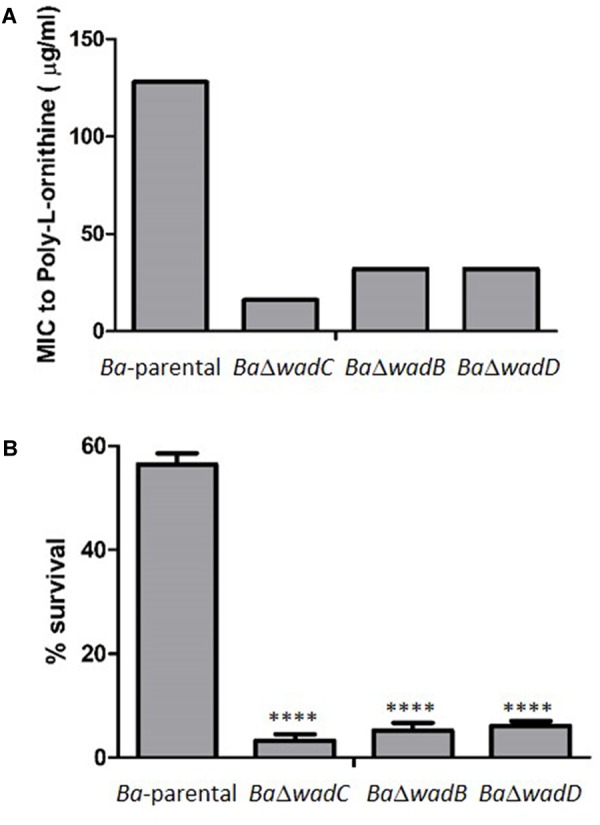
Dysfunction of *wadD* increases the sensitivity to poly-L-ornithine and non-immune sera. **(A)** Poly-L-ornithine MIC determined by the serial dilution method (results representative of three independent experiments in which *Ba*-parental, *Ba*Δ*wadC, Ba*Δ*wadB*, and *Ba*Δ*wadD* were assayed simultaneously). **(B)** Survival after incubation for 90 min in bovine non-immune serum (media ± standard error of technical triplicates). Means were compared by one-way ANOVA followed by Dunnett’s multiple comparisons test (^∗∗∗∗^*p* < 0.0001). Differences were significative between *wadC* and *wadB* mutants (*p* = 0.0023), between *wadC* and *wadD* mutants (*p* < 0.0001), but not between *wadB* and *wadD* mutants (*p* = 0.1958).

To test if the core defect displayed by *BaΔwadD* affected resistance to polycationic peptides, we used poly-L-ornithine, a mildly bactericidal cationic peptide and the two known core mutants *BaΔwadB* and *BaΔwadC* plus the parental strain as controls. The results (**Figure 5A**) showed that *wadD* dysfunction brought about a sensitivity similar to that of *BaΔwadB* but inferior to that of *BaΔwadC*. These differences in sensitivity were not due to growth defects because *BaΔwadD* had a growth rate similar to that of *Ba*-parental, and experiments with the highly bactericidal lipopeptide polymyxin B confirmed the role of *wadD* (*BaΔwadD* MIC = 0.094 μg/ml *versus* MIC = 2 μg/ml for both the mutant complemented with wild-type *wadD* and *Ba-*parental).

S *brucellae* are resistant to the bactericidal action of normal serum, a property associated with both the *O*-PS hindrance to inner OM targets such as OMPs and the PAMP modifications of the core that reduce binding of the complement activators of the antibody-independent classical pathway (Conde-Álvarez et al., 2012; Gil-Ramírez et al., 2014; Fontana et al., 2016). We compared the sensitivity to newborn bovine and ovine serum of *Ba*-parental and *wadB, wadC*, or *wadD* mutants and observed that the three core mutants were more sensitive than *Ba*-parental. The effect was more remarkable for mutant *wadC* than for *wadD* or *wadB* mutants (**Figure 5B**).

### Dysfunction of *wadD* Generates Attenuation Detectable in the Chronic Phase in the Mouse Model

**FIGURE 6:**
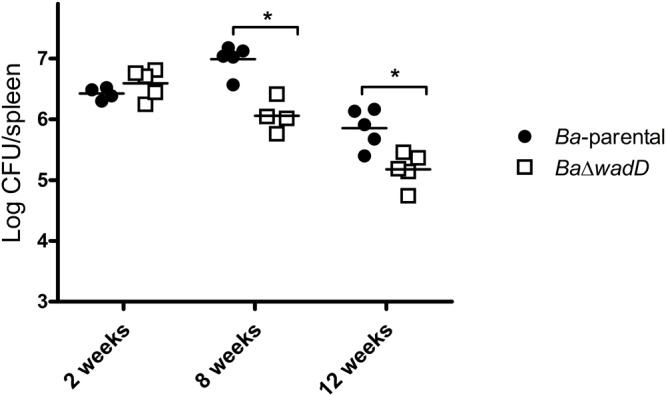
Mutants in *wadD* showed slight attenuation at late stage of infection. Spleen CFUs in infected BALB/c mice were counted at 2, 8, and 12 weeks after inoculation of 5 × 10^4^ CFU. Means were compared by Student’s *t* independent-samples test (^∗^*p* < 0.05). For mice infected with the parental strain the counts were 6.25 ± 0.38, 6.98 ± 0.24, and 5.85 ± 0.32, respectively, and for mice infected with the *wadD* mutant 6.56 ± 0.22, 6.28 ± 0.54, and 5.17 ± 0.27.

To analyze the role of *wadD* in virulence, we infected BALB/c mice (*n* = 5) with *Ba*Δ*wadD* or *Ba*-parental and compared the CFU/spleen at weeks 2, 8, and 12 (**Figure 6**). At weeks 8 and 12 post-infection, the CFU numbers of *Ba*Δ*wadD* were significantly lower than those of *Ba*-parental (*p* = 0.0003 and *p* = 0.0073, respectively), showing that *wadD* is required for full *Brucella* virulence in mice. This result is in line with previous observations with *wadB* and *wadC* mutants (Conde-Álvarez et al., 2012; Gil-Ramírez et al., 2014) and further confirms that an intact LPS core is necessary for virulence.

## Discussion

In this work, we have analyzed the role of ORFs BAB1_0114, BAB1_0417, BAB2_0693, BAB1_0932, BAB1_0607, BAB2_0105, BAB1_1620, BAB2_0133, BAB2_0135, and BAB1_0326 annotated as glycosyltransferases in the *B. abortus* genome. Our results indicate that mutants in these ORFs react similarly to the parental strain in the S and R-LPS fractions and suggest that, in the studied conditions and with the available techniques, they seem not to be required for the synthesis of a complete LPS. Interestingly, the last three ORFs have been shown to be controlled by *mucR*, a regulator of *Brucella* virulence. Although it has been reported that *B. abortus* and *B. melitensis mucR* mutants have a defect in the core LPS (Caswell et al., 2013; Mirabella et al., 2013), the glycosyltransferases responsible for this defect have not been identified. In this work we have shown that mutation of the *mucR*-regulated putative glycosyltransferases BAB2_0133, BAB2_0135, and BAB1_0326 (Caswell et al., 2013) does not affect the synthesis of the core, at least in the growth conditions tested. Nevertheless, since the expression of these genes seems to be repressed by *mucR* (Caswell et al., 2013), a single mutation in the ORF could not be sufficient for the complete clarification of their role in LPS synthesis and further work would be required.

We have also analyzed in detail the role of the hypothetical glycosyltransferase BAB1_1620, as it is regulated by the master two-component regulator BvrR/BvrS that controls *Brucella* virulence and the expression of surface components. According to our results, this ORF is not required for the synthesis of a complete LPS and is not implicated in surface-dependent characteristics that confer resistance to polycationic peptides or in virulence in the mouse model.

More interestingly, we report the identification of *wadD*, a gene encoding a previously unidentified glycosyltransferase involved in the synthesis of the core section not linked to the *O*-PS and thus, corroborate and extend previous work indicating that the *Brucella* LPS core is a branched structure that constitutes a steric impairment preventing the elements of the innate immune system to fight against *Brucella* (Conde-Álvarez et al., 2012; Kubler-Kielb and Vinogradov, 2013; Gil-Ramírez et al., 2014; Fontana et al., 2016), and contribute to *Brucella* virulence.

The discovery of genes *wadC* and *wadB*, involved in the synthesis of the lateral branch not linked to the *O*-PS, was critical for the understanding of the structure and the role of the core section in virulence. It has been clearly demostrated that in a *wadC* mutant, the complete core lateral branch is absent because this mutant cannot incorporate the mannose residue that is the depart of the lateral branch and it links to the lipid A-core section (Conde-Álvarez et al., 2012; Fontana et al., 2016). In accordance, deletion of *wadC* results in higher sensitivity to polycationic peptides and complement, better recognition by the CD14–MD2–TLR4 receptor complex, maturation of dendritic cells, secretion of pro-inflammatory cytokines (including Th1-type cytokines IL-12 and IFN-γ), and attenuation in mice (Conde-Álvarez et al., 2012; Fontana et al., 2016). A *wadB* mutant is also more sensitive to elements of the innate immune system and shows attenuation in mice, although not to the levels of the *wadC* mutant (Gil-Ramírez et al., 2014). As we show here, disruption of *wadD* in *B. abortus* leads to a S strain with a core defect less severe than that of the *wadC* mutant, more sensitive to polycationic peptides and normal serum than the parental strain and attenuated in the murine model. Interestingly, its sensitivity to polycationic peptides is similar to that of the *wadB* mutant (Gil-Ramírez et al., 2014) and not as strong as that of the *wadC* mutant (Conde-Álvarez et al., 2012), that has lost the complete branch, and its role in virulence became apparent already at 8 weeks post-infection.

The resistance to polycationic peptides and the bactericidal action of normal serum of mutant *wadD* strongly suggest a role in thwarting some effectors of innate immunity and that this could be manifested in the early stages of infection. However, to observe these effects would depend on the virulence model used. Indeed, the results obtained in an *in vitro* test, where the bacteria are put directly in contact with the polycationic peptides or with the serum can not be completely extrapolated to all situations in the *in vivo* model where other factors apart from polycationic peptides and complement are clearly taking part during the infection process. Still, the mouse model is the only well-characterized laboratory model for *Brucella* virulence studies and requires that mice are inoculated by the IP route. By this route, bacteria are not in contact with polycationic peptides or serum proteins at the beginning of the infection process as they are taken up and transported to the spleen rapidly. Nevertheless, the attenuation observed for *Brucella* LPS core mutants is caused by an early activation of innate immunity, as we have proved before (Conde-Álvarez et al., 2012; Fontana et al., 2016).

Our results would be compatible with the loss of one or few glucosamine residues in the lateral branch of the *wadD* mutant, and with the fact that removal of these residues would cause an increase in overall negative charge of the remaining LPS inner section that will facilitate the binding of polycationic peptides.

According to chemical studies performed in *B. melitensis*, the core lateral branch contains a mannose and four glucosamines residues assambled as follows: β-D-Glc*p*N-(1 → 6)-β-D-Glc*p*N-(1 → 4)-[β-D-Glc*p*N-(1 → 6)]-β-D-Glc*p*N-(1 → 3)-α-D-Man*p*-(1 → 5) (**Figure 1**). Taking into account that *wadC, wadB*, and *wadD* are perfectly conserved in *B. melitensis* and *B. abortus*, and since WadC adds the mannose (Conde-Álvarez et al., 2012; Fontana et al., 2016), in all likelihood the four glucosamines should be added by WadB (Gil-Ramírez et al., 2014) and WadD. These glucosamines are bound to each other by β-(1 → 6), or β-(1 → 4) links, and the one bound to mannose by β-(1 → 3) is also linked to two glucosamine residues, both in β-(1 → 6) and β-(1 → 4). If WadB and WadD are the only glycosyltransferases involved in the assembly of the glucosamine tetrasaccharide and its binding to the mannose residue, one of them (or both) could be multi-fiunctional and thus able to add sugars in different linkage. Most glycosyltransferase enzymes involved in lipooligosaccharide (LOS) or LPS core biosynthesis are responsible for one type of sugar addition onto the growing chain (Raetz and Whitfield, 2002). However, some bacterial glycosyltransferase enzymes of the GT-2 family, to which WadD belongs, can be multi-functional and are characterized by the presence of tandems of two active domains (DXD) on one polypeptide, as is the case of Lgt3, responsible for the addition of three glucoses with different linkages [β-(1-3), β-(1-4), and β-(1-6)] onto the inner core of *Moraxella catarrhalis* LOS (Coutinho et al., 2003; Luke-Marshall et al., 2013). Interestingly, WadD from *B. melitensis, B. abortus*, and all the orthologs in the other *Brucella* spp. conserves two DXD domains, opening the door to the possibility of a bi-functional role for this glycosyltransferase (**Supplementary Figure S5**). This DXD domain is not present in WadB. Nevertheless, the understanding of the particular role of each glycosyltransferase in the linkage of the different glucosamines to form the pentasaccharide (glucosamine tetrasaccharide bound to mannose) would require the elucidation of the core chemical structure of *wadB* and *wadD* mutants.

Contrary to most of the genes encoding glycosyltransferases implicated in the synthesis of the LPS, that are clustered in the same or related regions of the *Brucella* genome (Vemulapalli et al., 2000; Monreal et al., 2003; Rajashekara et al., 2004, 2008; González et al., 2008), *wadC, wadB*, and *wadD* (BAB1_1522, BAB1_0351, and BAB1_0953, respectively), although all situated in chromosome I, are isolated and surrounded by other ORFs apparently not related to LPS synthesis. This, and the fact that some other genes involved in the synthesis of the core (*manBcore* and *manCcore*) are situated in chromosome II (Monreal et al., 2003; González et al., 2008), makes even more intriguing the identification of genes needed for the synthesis of *Brucella* core LPS and its lateral branch. Thus, although we think all glycosyltransferases have been identified, we can not rule out that other glycosyltransferases could be required for the assembly of the pentasaccharide that forms the core lateral branch.

In a chemical characterization of the core LPS previously performed in a *B. melitensis* strain different from the one used in our studies, a glucose residue was found linked to the mannose that is the depart of the lateral branch, and, if this were the case, a new glycosyltransferase could be needed (Kubler-Kielb and Vinogradov, 2013). However, it should be taken into account that the LPS extraction method and the *B. melitensis* biovar used for the determination of the core structure in this experiement were different from those used in our genetic and biochemical studies (**Figure 1**). It is important to notice that the chemical structure we discuss in **Figure 1** has been elucidated in the same *B. melitensis* strain where the *wadC* gene was mutated (Fontana et al., 2016) and, in this case, no glucose residues were detected. The fact that, as discussed above, *wadC, wadB*, and *wadD* are perfectly conserved in *B. melitensis*, reinforces the interpretation of our results, and the idea that the glycosyltransferases encoded by the last two genes would be involved in the assembly of glucosamine residues.

Nevertheless, we should consider that, although the phenotype of *wadC* mutant in *B. melitensis* and *B. abortus* is similar (Conde-Álvarez et al., 2012; Fontana et al., 2016), previous results suggest that there could exist differences in the structure of the core in these two *Brucella* spp., since they react differently with MoAbs against core epitopes (González et al., 2008). Moreover, we have already seen that some of the studied ORFs (and discarded in our first screening since they reacted as the parental strain in the rough and smooth LPS fractions) present differences between *B. abortus* and *B. melitensis* (**Supplementary Table S2**). Thus, we could not discard them as the responsible for these differences. To understand the final role of *wadB, wadC*, and *wadD*, it would be necessary to analyze and compare the chemical structure of the core section in mutants in these genes in both spp.

## Ethics Statement

Female BALB/c mice (ENVIGO, Harlan) were kept in cages with water and food *ad libitum* under P3 biosafety conditions in the facilities of CIMA (registration code ES31 2010000132) or CITA (registration code ES502970012005) 2 weeks before and during the experiments. The procedures were in accordance with the current European (directive 86/609/EEC) and Spanish (RD 53/2013) legislations, supervised by the Animal Welfare Committee of the University of Navarra or CITA, and authorized by “Gobierno de Navarra” (protocol number 134-14) or “Gobierno de Aragón” (protocol number R108/2009).

## Author Contributions

MI, RC-Á, IM, and MS-B conceived the study. MS-B, YG-R, AZ-R, EM-G, MM, PM, AC, and MZ carried out the experimental work. MI, IM, RC-Á, and MS-B wrote the paper. All authors participated in the presentation and discussion of the results.

## Conflict of Interest Statement

The authors declare that the research was conducted in the absence of any commercial or financial relationships that could be construed as a potential conflict of interest.

## Footnotes

1 http://www.bacterio.net/-allnamesac.html

2 http://www.genome.jp/kegg/

3 http://blast.ncbi.nlm.nih.gov/Blast.cgi

4 http://www.ebi.ac.uk/Tools/msa/clustalo

5 http://www.ebi.ac.uk/

6 http://www.cazy.org

7 http://bioinfo.ut.ee/primer3-0.4.0/

8 http://www.cazy.org
