# Supplementary material for: WadD, a New Brucella Lipopolysaccharide Core Glycosyltransferase Identified by Genomic Search and Phenotypic Characterization
Source: Front Microbiol. 2018 Sep 27;9:2293. doi: 10.3389/fmicb.2018.02293 (PMC6171495; doi:10.3389/fmicb.2018.02293)
Supplement: Supplementary file 1 [file Data_Sheet_1.PDF]

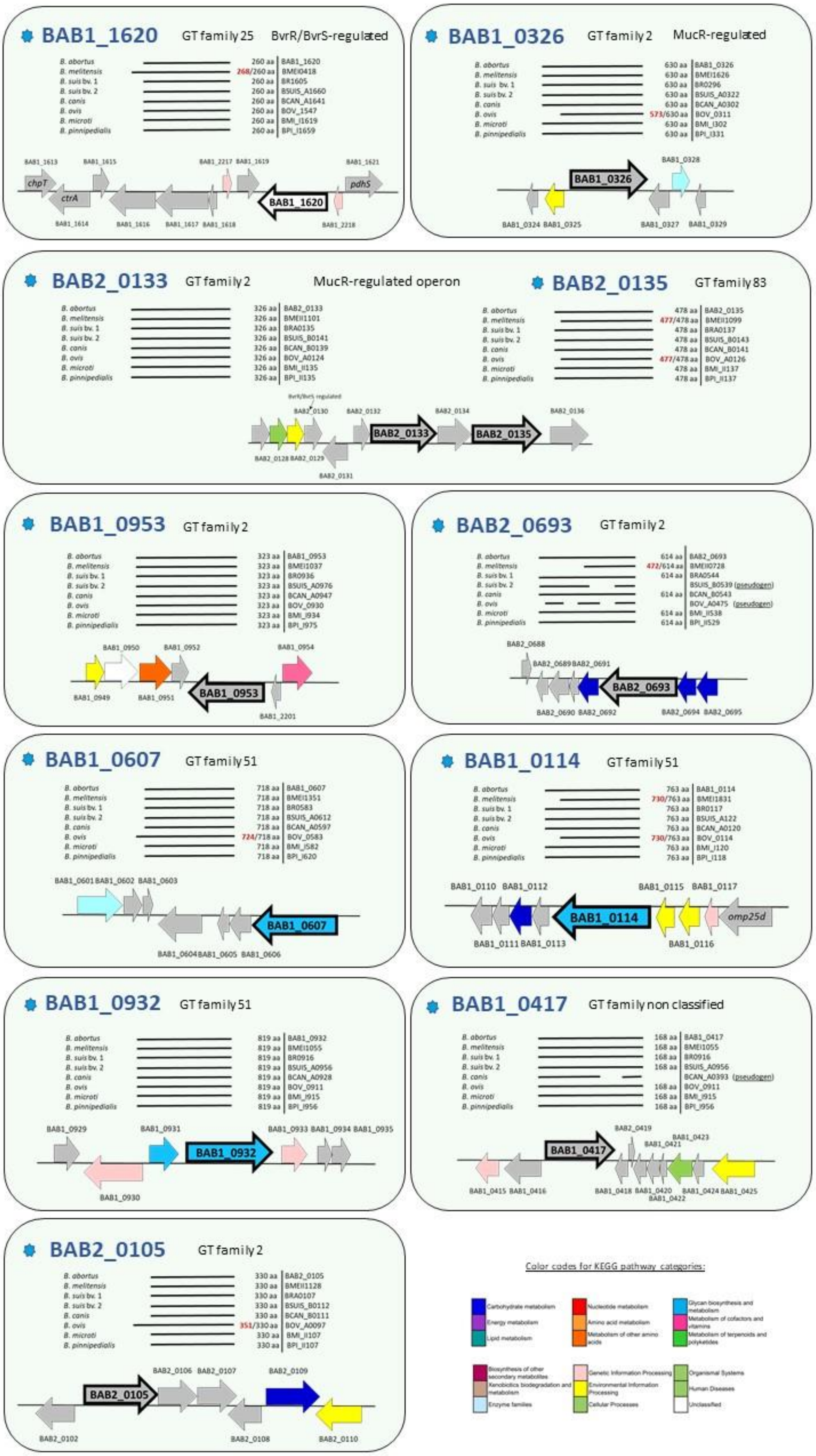
BAB1_1620
GT family 25
BvrR/BvrS-regulated
BAB1_0326
GT family 2
MucR-regulated
BAB2_0133
GT family 2
MucR-regulated operon
BAB2_0135
GT family 83
BAB1_0953
GT family 2
BAB2_0693
GT family 2
BAB1_0607
GT family 51
BAB1_0114
GT family 51
BAB1_0932
GT family 51
BAB1_0417
GT family non classified
BAB2_0105
GT family 2
Color codes for KEGG pathway categories:
Carbohydrate metabolism
Nucleotide metabolism
Glycan biosynthesis and metabolism
Energy metabolism
Amino acid metabolism
Metabolism of cofactors and vitamins
Lipid metabolism
Metabolism of other amino acids
Metabolism of terpenoids and polyketides
Biosynthesis of other secondary metabolites
Genetic Information Processing
Organismal Systems
Xenobiotics biodegradation and metabolism
Environmental Information Processing
Human Diseases
Enzyme families
Cellular Processes
Unclassified

Figure S1. ORFs not previously described as encoding hypothetical glycosyltransferases in *B. abortus* 2308, presence in other *Brucella* species, genetic regions, family to which they belong to and regulation. *B. melitensis* 16M; *B. suis* 1330 (biovar 1); *B. suis* ATCC 23445 (biovar 2); *B. canis* ATCC 23365; *B. ovis* ATCC 25840; *B. microti* CCM 4915 and *B. pinnipedialis* B2/94.
