# Supplementary material for: WadD, a New Brucella Lipopolysaccharide Core Glycosyltransferase Identified by Genomic Search and Phenotypic Characterization
Source: Front Microbiol. 2018 Sep 27;9:2293. doi: 10.3389/fmicb.2018.02293 (PMC6171495; doi:10.3389/fmicb.2018.02293)
Supplement: Supplementary file 2 [file Data_Sheet_2.PDF]

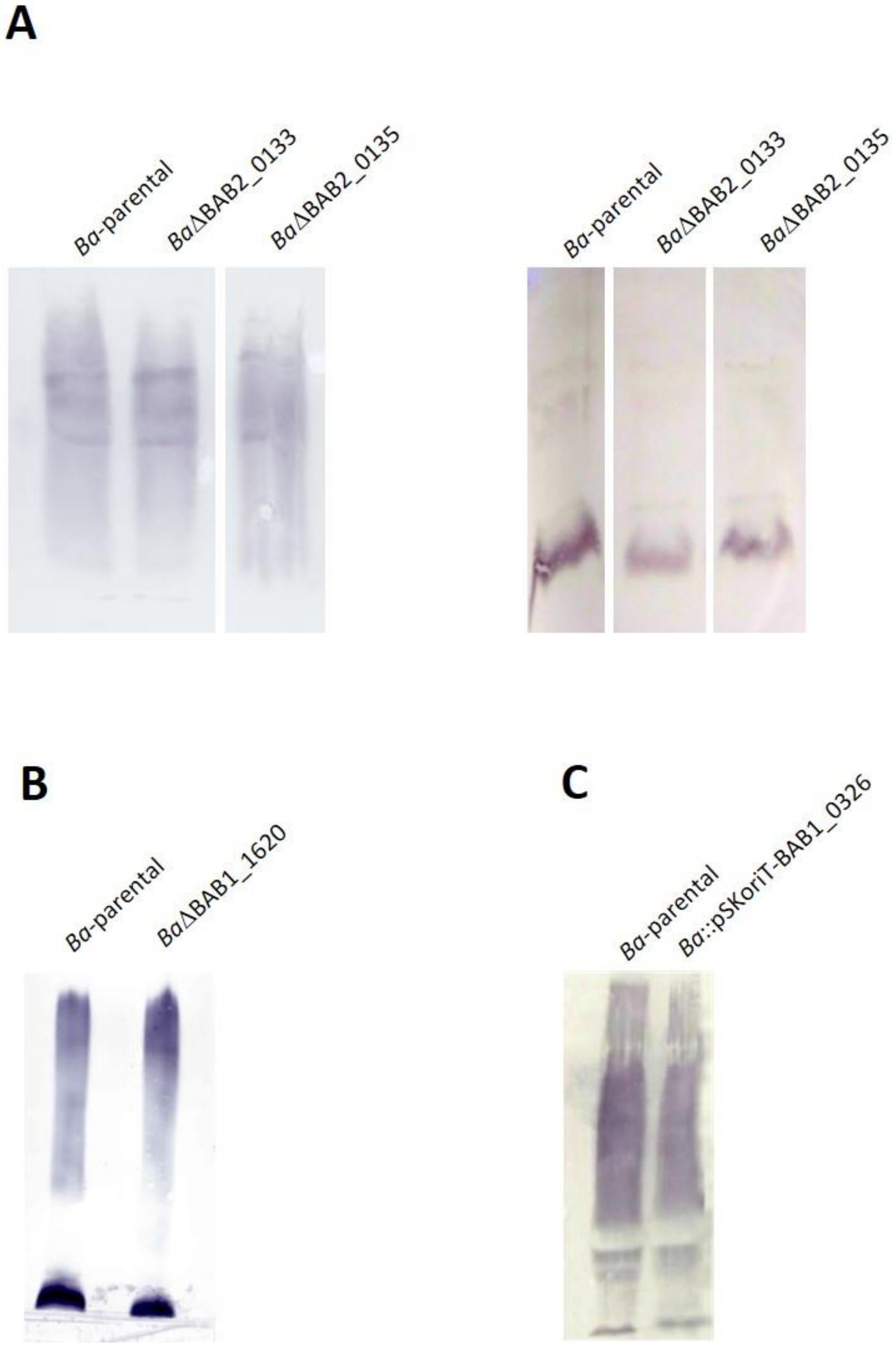

Figure S2. The hypothetical glycosyltransferases controlled by MucR or BvrR/S are not required for the synthesis of a complete core LPS. **A.** Western-blot analysis of LPS extracts of mutants in BAB2_0133 and BAB2_0135 with polyclonal serum against S-*Brucella* (left panel) and with monoclonal anti-core antibody A68/24G12/A08 (right panel). **B.** Western-blot analysis of LPS of mutant in BAB1_1620 with monoclonal anti-core antibody A68/24G12/A08. **C.** Western-blot analysis of the LPS of an insertion mutant in BAB1_0326 with polyclonal serum anti-S-*Brucella*.
