# Supplementary material for: WadD, a New Brucella Lipopolysaccharide Core Glycosyltransferase Identified by Genomic Search and Phenotypic Characterization
Source: Front Microbiol. 2018 Sep 27;9:2293. doi: 10.3389/fmicb.2018.02293 (PMC6171495; doi:10.3389/fmicb.2018.02293)
Supplement: Supplementary file 3 [file Data_Sheet_3.PDF]

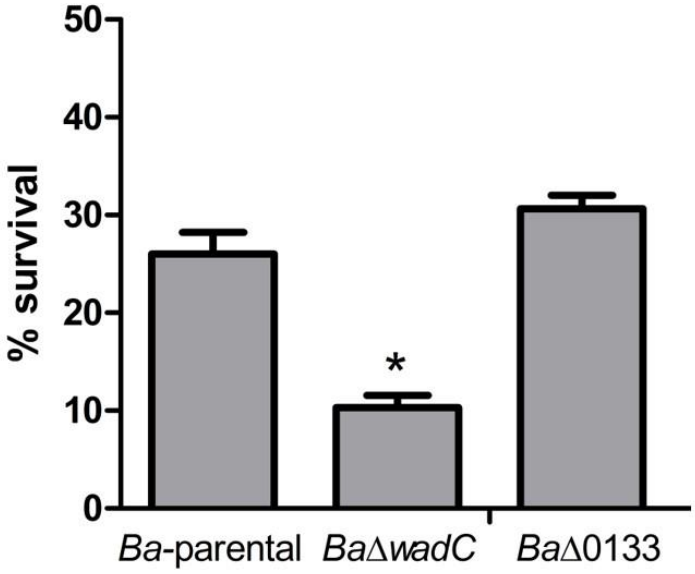

Figure S3. Mutant in BAB2_0133 was as sensitive to the lytic action of complement present in bovine serum as the parental strain. Survival after incubation for 90 minutes in bovine non-immune serum (media ± standard error of technical triplicates). Means were compared by one-way ANOVA followed by Dunnett´s multiple comparisons test (∗$p < 0.05$). *Ba*Δ*wadC* mutant was used as a control.
