# Supplementary material for: WadD, a New Brucella Lipopolysaccharide Core Glycosyltransferase Identified by Genomic Search and Phenotypic Characterization
Source: Front Microbiol. 2018 Sep 27;9:2293. doi: 10.3389/fmicb.2018.02293 (PMC6171495; doi:10.3389/fmicb.2018.02293)
Supplement: Supplementary file 4 [file Data_Sheet_4.PDF]

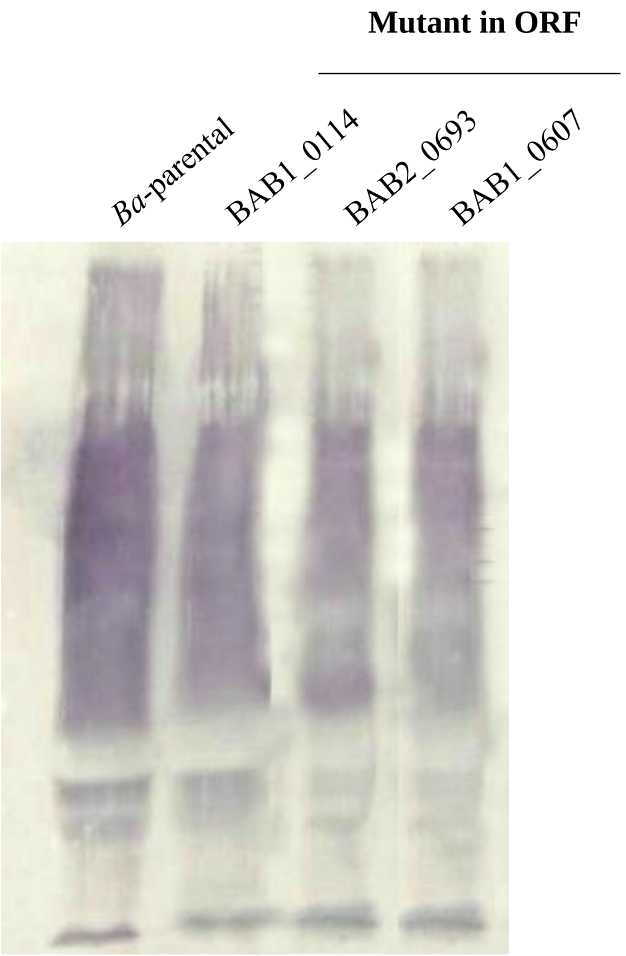

Figure S4. Mutation in ORFs BAB1_0114, BAB2_0693 or BAB1_0607 does not generate a truncated LPS. Western blot analysis of LPS extracts with a polyclonal serum against S-*Brucella*.
