# Supplementary material for: WadD, a New Brucella Lipopolysaccharide Core Glycosyltransferase Identified by Genomic Search and Phenotypic Characterization
Source: Front Microbiol. 2018 Sep 27;9:2293. doi: 10.3389/fmicb.2018.02293 (PMC6171495; doi:10.3389/fmicb.2018.02293)
Supplement: Supplementary file 5 [file Data_Sheet_5.PDF]

```
B. abortus           MPIFKIIIATTTRNRPKMLINLYKSLGDLEIPSNIDVEFLIVENNRTSTSESWLHEIRS
B. melitensis        MPIFKIIIATTTRNRPKMLINLYKSLGDLEIPSNIDVEFLIVENNRTSTSESWLHEIRS
B. suis bv. 1        MPIFKIIIATTTRNRPKMLINLYKSLGDLEIPSNIDVEFLIVENNRTSTSESWLHEIRS
B. suis bv. 2        MPIFKIIIATTTRNRPKMLINLYKSLGDLEIPSNIDVEFLIVENNRTSTSESWLHEIRS
B. suis bv. 5        MPIFKIIIATTTRNRPKMLINLYKSLGDLEIPSNIDVEFLIVENNRTSTSESWLHEIRS
B. ovis              MPIFKIIIATTTRNRPKMLINLYKSLGDLEIPSNIDVEFLIVENNRTSTSESWLHEIRS
B. canis             MPIFKIIIATTTRNRPKMLINLYKSLGDLEIPSNIDVEFLIVENNRTSTSESWLHEIRS
B. microti           MPIFKIIIATTTRNRPKMLINLYKSLGDLEIPSNIDVEFLIVENNRTSTSESWLHEIRS
B. pinnipedialis     MPIFKIIIATTTRNRPKMLINLYKSLGDLEIPSNIDVEFLIVENNRTSTSESWLHEIRS
B. vulpis            MPIFKIIIATTTRNRPKMLINLYKSLSDLEIPSNIDVEFLIVENNRTSTSENWLHEIRC
B. ceti              MPIFKIIIATTTRNRPKMLINLYKSLGDLEIPSNIDVEFLIVENNRTSTSESWLHEIRS
B. inopinata         MPIFKIIIATTTRNRPKMLINLYKSLSDLEIPSNIDVEFLIVENNRTSTSENWLHEIRS
B. inopinata BO2     MPIFKIIIATTTRNRPKMLINLYKSLSDLEIPSNIDVEFLIVENNRTSTSENWLHEIRS
NF2653 (Austr.)      MPIFKIIIATTTRNRPKMLINLYKSLSDLEIPSNIDVEFLIVENNRTSTSENWLHEIRS
09RB8471(frog)       MPIFKIIIATTTRNRPKMLINLYKSLSDLEIPSNIDVEFLIVENNKTSTSENWLHEIRS
                                               *                  *     *      *
```

```
B. abortus           SISPSAVVYILETSIGISCARNRALDYAQEAGADFLAFVDDDEFVEPDWLKQLFAEQQRR
B. melitensis        SISPSAVVYILETSIGISCARNRALDYAQEAGADFLAFVDDDEFVEPDWLKQLFAEQQRR
B. suis bv. 1        SISPSAVVYILETSIGISCARNRALDYAQEAGADFLAFVDDDEFVEPDWLKQLFAEQQRR
B. suis bv. 2        SISPSAVVYILETSIGISCARNRALDYAQEAGADFLAFVDDDEFVEPDWLKQLFAEQQRR
B. suis bv. 5        SISPSAVVYILETSIGISCARNRALDYAQEAGADFLAFVDDDEFVEPDWLKQLFAEQQRR
B. ovis              SISPSAVVYILETSIGISCARNRALDYAQEAGADFLAFVDDDEFVEPDWLKQLFAEQQRR
B. canis             SISPSAVVYILETSIGISCARNRALDYAQEAGADFLAFVDDDEFVEPDWLKQLFAEQQRR
B. microti           SISPSAVVYILETSIGISCARNRALDYAQEAGADFLAFVDDDEFVEPDWLKQLFAEQQRR
B. pinnipedialis     SISPSAVVYILETSIDISCARNRALDYAQEAGADFLAFVDDDEFVEPDWLKQLFAEQQRR
B. vulpis            SISPSAVVYILETSIGISCARNRALDYAQEAGADFLAFVDDDEFVEPDWLKQLFAEQQRR
B. ceti              SISPSAVVYILETSIDISCARNRALDYAQEAGADFLAFVDDDEFVEPDWLKQLFAEQQRR
B. inopinata         SISSSAVVYILETSIGISCARNRALDYAQEAGADFLAFVDDDEFVEPDWLKQLFAEQQRR
B. inopinata BO2     SISPSAVVYILETSIGISCARNRALDYAQEAGADFLAFVDDDEFVEPDWLKQLFAEQQRR
NF2653 (Austr.)      SISPSAVVYILETSIGISCARNRALDYAQEAGADFLAFVDDDEFVEPDWLKQLFAEQQRR
09RB8471(frog)       SIPSSAVVYILETSIGISCARNRALDYAQEASADFLAFVDDDEFVEPDWLKQLFAEQQRR
                       **           *               *
```

```
B. abortus           DLDLVGSPVRPVPQNSKLSLWQRFVWSGVERNGTRAEDRARRKWQENKADTIKIATGSWL
B. melitensis        DLDLVGSPVRPVPQNSKLSLWQRFVWSGVERNGTRAEDRARRKWQENKADTIKIATGSWL
B. suis bv. 1        DLDLVGSPVRPVPQNSKLSLWQRFVWSGVERNGTRAEDRARRKWQENKADTIKIATGSWL
B. suis bv. 2        DLDLVGSPVRPVPQNSKLSLWQRFVWSGVERNGTRAEDRARRKWQENKADTIKIATGSWL
B. suis bv. 5        DLDLVGSPVRPVPQNSKLSLWQRFVWSGVERNGTRAEDRARRKWQENKADTIKIATGSWL
B. ovis              DLDLVGSPVRPVPQNSKLSLWQRFVWSGVERNGTRAEDRARRKWQENKADTIKIATGSWL
B. canis             DLDLVGSPVRPVPQNSKLSLWQRFVWSGVERNGTRAEDRARRKWQENKADTIKIATGSWL
B. microti           DLDLVGSPVRPVPQNSKLSLWQRFVWSGVERNGTRAEDRARRKWQENKADTIKIATGSWL
B. pinnipedialis     DLDLVGSPVRPVPQNSKLSLWQRFVWSGVERNGTRAEDRARRKWQENKADTIKIATGSWL
B. vulpis            DLDLVGSPVRPVPQNSKLSLWQRFVWSGVERNGTRAEDRARRKWQENKADTIKIATGSWL
B. ceti              DLDLVGSPVRPVPQNSKLSLWQRFVWSGVERNGTRAEDRARRKWQENKADTIKIATGSWL
B. inopinata         DLDLVGSPVRPVPQNSKLSLWQRFVWSGVERNGTRAEDRARRKWQENKADTIKIATGSWL
B. inopinata BO2     DLDLVGSPVRPVPQNSKLSLWQRFVWSGVERNGTRAEDRARRKWQENKADTIKIATGSWL
NF2653 (Austr.)      DLDLVGSPVRPVPQNSKLSLWQRFVWSGVERNGTRAEDRARRKWQENKADTIKIATGSWL
09RB8471(frog)       DLDLVGSPVRPVPQNSKLSLWQRFVWSGVERNGTRAEDRARRKWQENKADTIKIATGSWL
```

```
B. abortus           GRIDFFRRTGLRFDSKLGLTGGEDWNLWLEAKKLGAKTGWAPDAIVYETVPYCRISFSYH
B. melitensis        GRIDFFRRTGLRFDSKLGLTGGEDWNLWLEAKKLGAKTGWAPDAIVYETVPYCRISFSYH
B. suis bv. 1        GRIDFFRRTGLRFDSKLGLTGGEDWNLWLEAKKLGAKTGWAPDAIVYETVPYCRISFSYH
B. suis bv. 2        GRIDFFRRTGLRFDSKLGLTGGEDWNLWLEAKKLGAKTGWAPDAIVYETVPYCRISFSYH
B. suis bv. 5        GRIDFFRRTGLRFDSKLGLTGGEDWNLWLEAKKLGAKTGWAPDAIVYETVPYCRISFSYH
B. ovis              GRIDFFRRTGLRFDSKLGLTGGEDWNLWLEAKKLGAKTGWAPDAIVYETVPYCRISFSYH
B. canis             GRIDFFRRTGLRFDSKLGLTGGEDWNLWLEAKKLGAKTGWAPDAIVYETVPYCRISFSYH
B. microti           GRIDFFRRTGLRFDSKLGLTGGEDWNLWLEAKKLGAKTGWAPDAIVYETVPYCRISFSYH
B. pinnipedialis     GRIDFFRRTGLRFDSKLGLTGGEDWNLWLEAKKLGAKTGWAPDAIVYETVPYCRISFSYH
B. vulpis            GRIDFFRRTGLRFDSKLGLTGGEDWNLWLEAKKLGAKTGWAPDAIVYETVPYCRISFSYH
B. ceti              GRIDFFRRTGLRFDSKLGLTGGEDWNLWLEAKKLGAKTGWAPDAIVYETVPYCRISFSYH
B. inopinata         GRIDFFRRTGLRFDSKLGLTGGEDWNLWLEAKKHGAKTGWAPDAIVYETVPHCRISFSYH
```

```
B. inopinata BO2  GRIDFFRRTGLRFDSKLGLTGGEDWNLWLEAKKLGAKTGWAPDAIVYETVPHCRISFSYH
NF2653 (Austr.)   GRIDFFRRTGLRFDSKLGLTGGEDWNLWLEAKKLGAKTGWAPDAIVYETVPYCRISFSYH
09RB8471(frog)    GRIDFFRKTGLRFDSKLGLTGGEDWNLWLEAKKLGAKTGWAPDAIVYETVPYCRISFSYH
                         *                         *                 *


B. abortus        FRRNRDHNATEFTLLYSKSPRRAWMRLPSRILSRVWKLLTAILTLPFKGGQALISLAMAL
B. melitensis     FRRNRDHNATEFTLLYSKSPRRAWMRLPSRILSRVWKLLTAILTLPFKGGQALISLAMAL
B. suis bv. 1     FRRNRDHNATEFTLLYSKSPRRAWMRLPSRILSRVWKLLTAILTLPFKGGQALISLAMAL
B. suis bv. 2     FRRNRDHNATEFTLLYSKSPRRAWMRLPSRILSRVWKLLTAILTLPFKGGQALISLAMAL
B. suis bv. 5     FRRNRDHNATEFTLLYSKSPRRAWMRLPSRILSRVWKLITAILTLPFKGGQALISLAMAL
B. ovis           FRRNRDHNATEFTLLYSKSPRRAWMRLPSRILSRVWKLLTAILTLPFKGGQALISLAMAL
B. canis          FRRNRDHNATEFTLLYSKSPRRAWMRLPSRILSRVWKLLTAILTLPFKGGQALISLAMAL
B. microti        FRRNRDHNATEFTLLYSKSPRRAWMRLPSRILSRVWKLLTAILTLPFKGGQALISLAMAL
B. pinnipedialis  FRRNRDHNATEFTLLYSKSPRRAWMRLPSRILSRVWKLLTAILTLPFKGGQALISLAMAL
B. vulpis         FRRNRDHNATEFTLLYSKSPRRAWMRLPSRILSRVWKLLTAILTLPFKGGQALISLAMAL
B. ceti           FRRNRDHNATEFTLLYSKSPRRAWMRLPSRILSRVWKLLTAILTLPFKGGQALISLAMAL
B. inopinata      FRRNRDHNATEFTLLYSKSPRRAWMRLPSRILSRVWKLLTAILTLPFRGGQALISLAMAL
B. inopinata BO2  FRRNRDHNATEFTLLYSKSPRRAWMRLPSRILSRVWKLLTAILTLPFRGGQALISLAMAL
NF2653 (Austr.)   FRRNRDHNATEFTLLYSKSPRRAWMRLPSRILSRVWKLLTAILTLPFKGGQALISLAMAL
09RB8471(frog)    FRRNRDHNATEFTLLYSKSPRRAWMRLPSRILSRVWKLLTAILTLPFKGGRLSSRWRWLW
                                                                 *  **********


B. abortus        GGIVGLVQACCGKQQLHYKETTGS
B. melitensis     GGIVGLVQACCGKQQLHYKETTGS
B. suis bv. 1     GGIVGLVQACCGKQQLHYKETTGS
B. suis bv. 2     GGIVGLVQACCGKQQLHYKETTGS
B. suis bv. 5     GGIVGLVQACCGKQQLHYKETTGS
B. ovis           GGIVGLVQACCGKQQLHYKETTGS
B. canis          GGIVGLVQACCGKQQLHYKETTGS
B. microti        GGIVGLVQACCGKQQLHYKETTGS
B. pinnipedialis  GGIVGLVQACCGKQQLHYKETTGS
B. vulpis         GGIVGLVQACCGKQQLHYKETTGS
B. ceti           GGIVGLVQACCGKQQLHYKETTGS
B. inopinata      GGIVGLVQACCGKQQLHYKETTGS
B. inopinata BO2  GGIVGLVQACCGKQQLHYKETTGS
NF2653 (Austr.)   GGIVGLVQACCGKQQLHYKETTGS
09RB8471(frog)    EELSGWSRPAAENSSFIIRKQPAL
                  **** *******************
```

Figure S5. Alignment of the protein encoded by *wadD* in *B. abortus* (2308); *B. melitensis* (strain 16M), *B. suis* bv. 1 (strain 1330); *B. suis* bv. 2 (strain ATCC 23445 or Thomsen); *B. suis* bv. 5 (strain 513); *B. ovis* (strain ATCC 25840); *B. canis* (strain ATCC 23365); *B. microti* (strain CCM 4915); *B. pinnipedialis* (strain B2/94); *B. vulpis*; *B. ceti*; *B. inopinata* (strain BO1); *B. inopinata* BO2 (*Brucella innopinata*-like BO2); NF2653 (Austr.) (*Brucella* spp. NF 2653, Australian isolate) and *Brucella* spp. 09RB8471 isolated from amphibians. The underlined spp. have changes in the aminoacid sequence when comparing with *B. abortus*. The changes are signalled in grey.
