# Supplementary material for: WadD, a New Brucella Lipopolysaccharide Core Glycosyltransferase Identified by Genomic Search and Phenotypic Characterization
Source: Front Microbiol. 2018 Sep 27;9:2293. doi: 10.3389/fmicb.2018.02293 (PMC6171495; doi:10.3389/fmicb.2018.02293)
Supplement: Supplementary file 6 [file Data_Sheet_6.pdf]

Table S1. Bacterial strains and plasmids.

Strain	Characteristics	Reference
Brucella abortus		
Ba-parental	*B. abortus* wild type, virulent biotipe 1, smooth LPS, Nal^R spontaneous mutant of strain *B. abortus* 2308W (previously called 2308 (Conde-Álvarez et al., 2012, 2013, 2018; Fontana et al., 2016; Gil-Ramírez et al., 2014), renamed after 2308W (Suarez-Esquivel et al., 2016; Zúñiga-Ripa et al., 2018).	(Sangari and Agüero, 1991)
Ba::pSKoriT-BAB1_0326	*Ba*-parental insertion mutant in BAB1_0326$_{::261\text{-}383}$	This work
Ba::pSKoriT-BAB1_0417	*Ba*-parental insertion mutant in BAB1_0417$_{::37\text{-}134}$	This work
Ba:: pSKoriT-BAB1_0953	*Ba*-parental insertion mutant in BAB1_0953$_{::112\text{-}230}$	This work
Ba::pJQK-BAB1_0932	*Ba*-parental insertion mutant in BAB1_0932$_{::354\text{-}493}$	This work
Ba::pJQK-BAB1_0114	*Ba*-parental insertion mutant in BAB1_0114$_{::248\text{-}407}$	This work
Ba::pJQK-BAB2_0693	*Ba*-parental insertion mutant in BAB2_0693$_{::249\text{-}386}$	This work
Ba::pJQK-BAB1_0607	*Ba*-parental insertion mutant in BAB1_0607$_{::278\text{-}427}$	This work
*Ba*ΔBAB2_0133	*Ba*-parental deletion mutant in BAB2_0133$_{\Delta 38\text{-}299}$	This work
*Ba*ΔBAB2_0135	*Ba*-parental deletion mutant in BAB2_0135$_{\Delta 40\text{-}441}$	This work
*Ba*ΔBAB2_0105	*Ba*-parental deletion mutant in BAB2_0105$_{\Delta 33\text{-}307}$	This work
*Ba*ΔBAB1_1620	*Ba*-parental deletion mutant in BAB1_1620$_{\Delta 23\text{-}241}$	This work
*Ba*Δ*wadD*	*Ba*-parental deletion mutant in BAB1_0953$_{\Delta 50\text{-}281}$	This work
*Ba*Δ*wadD*::Tn7-PwadD	*Ba*Δ*wadD* complemented strain with miniTn7T Km^R harbouring BAB1_0953 complete ORF with its own promoter.	This work
*Ba*Δ*wadB*	*Ba*-parental LPS core mutant	(Gil-Ramírez et al., 2014)
*Ba*Δ*wadC*	*Ba*-parental LPS core mutant	(Conde-Álvarez et al., 2012)
*Ba*Δ*per*	*Ba*-parental O-PS mutant	(Martínez-Gómez et al., 2018)
Escherichia coli		
TOP10	F – lac/q Tn 10 (Tetr) mcrA Δ(mrr-hsdRMS-mcrBC) 80lacZΔM15. ΔlacX74 recA1alaD139 Δ(ara-leu)7697 galU galK rpsL endA1 nupG	Invitrogen
Stellar	F–, endA1, supE44, thi-1, recA1, relA1, gyrA96 phoA, Φ80d lacZΔ M15, Δ(lacZYA-argF) U169 Δ(mrr-hsdRMS-mcrBC), ΔmcrA, λ–	Clontech

Strain	Characteristics	Reference
S17 λ pir	Mating strain with plasmid RP4 inserted into the chromosome	(Simon et al., 1983)
PIR1	F-Δlac169 rpoS(Am) robA1 creC510 hsdR514 endA recA1 uidA(ΔMluI)::pir-116	Invitrogen
SM10 λpir-pTNS2	Plasmid encoding the information for Tn7 transposition in the right place. Amp^R 100 μg/ml	(Choi et al., 2005)
HB101- pRK2013	pRK2013 is a helper plasmid for conjugation. Km^R 35μg/ml	(Choi et al., 2005)

Plasmid	Characteristics	Reference
pCR2.1	Cloning vector, Km^R	Invitrogen
pJQKm	Derivate of pJQ200KS+. Suicide vector, Km^R Sac^S	(Scupham and Triplett, 1997)
pSKoriT	Derivative of pBluescript. Suicide vector, Km^R Sac^S	(Tibor et al., 2002)
pUC18 R6KT mini Tn7T Km^R	Broad host-range mini-Tn7 vector	(Llobet et al., 2009)
pMSB-1	1014 bp of *B. abortus* chromosomal DNA containing the BAB2_0133 deletion allele (Δ38-299), generated by PCR and cloned into pCR2.1.	This work
pMSB-2	*Bam*HI/*Xba*I fragment from pMSB-1 cloned into the corresponding sites of pJQKm.	This work
pMSB-5	295 bp of *B. abortus* chromosomal DNA containing the BAB1_0417 internal fragment, generated by PCR and cloned into pCR2.1.	This work
pMSB-6	BamHI/XbaI fragment from pMSB-5 cloned into the corresponding sites of pSKoriT.	This work
pMSB-7	368 bp of *B. abortus* chromosomal DNA containing the BAB1_0326 internal fragment, generated by PCR and cloned into pCR2.1.	This work
pMSB-8	353 bp of *B. abortus* chromosomal DNA containing the BAB2_0105 internal fragment, generated by PCR and cloned into pCR2.1.	This work
pMSB-9	*Bam*HI/*Xba*I fragment from pMSB-8 cloned into the corresponding sites of pSKoriT.	This work
pMSB-10	*Bam*HI/*Xba*I fragment from pMSB-7 cloned into the corresponding sites of pSKoriT.	This work
pMSB-11	358 bp of *B. abortus* chromosomal DNA containing the BAB1_0953 internal fragment, generated by PCR and cloned into pCR2.1.	This work

Plasmid	Characteristics	Reference
pMSB-12	*Bam*HI/*Xba*I fragment from pMSB-11 cloned into the corresponding sites of pSKoriT.	This work
pMSB-14	486 bp of *B. abortus* chromosomal DNA containing the BAB2_0135 internal fragment, generated by PCR and cloned into pCR2.1.	This work
pMSB-15	*Bam*HI/*Xba*I fragment from pMSB-14 cloned into the corresponding sites of pSKoriT.	This work
pMSB-16	416 bp of *B. abortus* chromosomal DNA containing the BAB2_0693 internal fragment, generated by PCR and cloned into pCR2.1.	This work
pMSB-17	481 bp of *B. abortus* chromosomal DNA containing the BAB1_0114 internal fragment, generated by PCR and cloned into pCR2.1.	This work
pMSB-19	449 bp of *B. abortus* chromosomal DNA containing the BAB1_0607 internal fragment, generated by PCR and cloned into pCR2.1.	This work
pMSB-20	352 bp of *B. abortus* chromosomal DNA containing the BAB2_0105 deletion allele (Δ33-307), generated by PCR and cloned into pCR2.1.	This work
pMSB-21	*Bam*HI/*Xba*I fragment from pMSB-19 cloned into the corresponding sites of pJQKm.	This work
pMSB-25	*Bam*HI/*Xba*I fragment from pMSB-16 cloned into the corresponding sites of pJQKm.	This work
pMSB-27	*Bam*HI/*Xba*I fragment from pMSB-20 cloned into the corresponding sites of pJQKm.	This work
pMSB-28	*Bam*HI/*Xba*I fragment from pMSB-17 cloned into the corresponding sites of pJQKm.	This work
pMSB-29	420 bp of *B. abortus* chromosomal DNA containing the BAB1_0932 internal fragment, generated by PCR and cloned into pCR2.1.	This work
pMSB-30	*Bam*HI/*Xba*I fragment from pMSB-29 cloned into the corresponding sites of pJQKm.	This work
pMSB-34	*Xba*I fragment of 479 bp from *B. abortus* chromosomal DNA containing the BAB1_0953 deletion allele (Δ50-281) cloned into the corresponding sites of pJQKm by InFusion HD Cloning System.	This work
pMSB-37	*Xba*I fragment of 472 bp from *B. abortus* chromosomal DNA containing the BAB2_0135 deletion allele (Δ40-441) cloned into the corresponding sites of pJQKm by InFusion HD Cloning System.	This work

Plasmid	Characteristics	Reference
pMSB-44	*Eco*RI fragment of 1771 bp from *B. abortus* Chromosomal DNA containing the BAB1_0953 complete allele and its own promoter, cloned into the corresponding sites of pUC18 R6KT miniTn7T Km^R by InFusion HD Cloning System.	This work
pYRI-16	885 bp of *B. abortus* chromosomal DNA containing the BAB1_1620 deletion allele (Δ23-241), generated by PCR and cloned into pCR2.1.	This work
pYRI-17	*Bam*HI/*Xba*I fragment from pMSB-19 cloned into the corresponding sites of pJQKm.	This work

References

Choi, K.-H., Gaynor, J. B., White, K. G., Lopez, C., Bosio, C. M., Karkhoff-Schweizer, R. R., et al. (2005). A Tn7-based broad-range bacterial cloning and expression system. *Nat. Methods* 2, 443–448. doi:10.1038/nmeth765.

Conde-Álvarez, R., Arce-Gorvel, V., Gil-Ramírez, Y., Iriarte, M., Grilló, M. J., Gorvel, J. P., et al. (2013). Lipopolysaccharide as a target for brucellosis vaccine design. *Microb. Pathog.* 58, 29–34. doi:10.1016/j.micpath.2012.11.011.

Conde-Álvarez, R., Arce-Gorvel, V., Iriarte, M., Manček-Keber, M., Barquero-Calvo, E., Palacios-Chaves, L., et al. (2012). The lipopolysaccharide core of *Brucella abortus* acts as a shield against innate immunity recognition. *PLoS Pathog.* 8, e1002675. doi:10.1371/journal.ppat.1002675.

Conde-Álvarez, R., Palacios-Chaves, L., Gil-Ramírez, Y., Salvador-Bescós, M., Bárcena-Varela, M., Aragón-Aranda, B., et al. (2018). Identification of *lptA, lpxE*, and *lpxO*, Three Genes Involved in the Remodeling of *Brucella* Cell Envelope. *Front. Microbiol.* 8, 2657. doi:10.3389/fmicb.2017.02657.

Fontana, C., Conde-Álvarez, R., Ståhle, J., Holst, O., Iriarte, M., Zhao, Y., et al. (2016). Structural studies of lipopolysaccharide defective mutants from *Brucella melitensis* identify a core oligosaccharide critical in virulence. *J. Biol. Chem.* 291, 7727–7741. doi:10.1074/jbc.M115.701540.

Gil-Ramírez, Y., Conde-Álvarez, R., Palacios-Chaves, L., Zúñiga-Ripa, A., Grilló, M.-J., Arce-Gorvel, V., et al. (2014). The identification of *wadB*, a new glycosyltransferase gene, confirms the branched structure and the role in virulence of the lipopolysaccharide core of *Brucella abortus*. *Microb. Pathog.* 73, 53–9. doi:10.1016/j.micpath.2014.06.002.

Llobet, E., March, C., Giménez, P., and Bengoechea, J. A. (2009). *Klebsiella pneumoniae* OmpA Confers Resistance to Antimicrobial Peptides. *Antimicrob. Agents Chemother.* 53, 298–302. doi:10.1128/AAC.00657-08.

Martínez-Gómez, E., Ståhle, J., Gil-Ramírez, Y., Zúñiga-Ripa, A., Zaccheus, M., Moriyón, I., et al. (2018). Genomic insertion of a heterologous acetyltransferase generates a new lipopolysaccharide antigenic structure in *Brucella abortus* and *Brucella melitensis*. *Front. Microbiol.* 9, 1092. doi:10.3389/FMICB.2018.01092.

Sangari, F., and Agüero, J. (1991). Mutagenesis of *Brucella abortus*: comparative efficiency of three transposon delivery systems. *Microb. Pathog.* 11, 443–6.

Scupham, A. J., and Triplett, E. W. (1997). Isolation and characterization of the UDP-glucose 4'-epimerase-encoding gene, *galE*, from *Brucella abortus* 2308. *Gene* 202, 53–59. doi:10.1016/S0378-1119(97)00453-8.

Simon, R., Priefer, U., and Pühler, A. (1983). A Broad Host Range Mobilization System for In Vivo Genetic Engineering: Transposon Mutagenesis in Gram Negative Bacteria. *Bio/Technology* 1, 784–791. doi:10.1038/nbt1183-784.

Suarez-Esquivel, M., Ruiz-Villalobos, N., Castillo-Zeledon, A., Jimenez Rojas, C., Roop, R., Comerci, D., et al. (2016). *Brucella abortus* Strain 2308 Wisconsin Genome: Importance of the Definition of Reference Strains. doi:10.3389/fmicb.2016.01557.

Tibor, A., Wansard, V., Bielartz, V., Delrue, R.-M., Danese, I., Michel, P., et al. (2002). Effect of *omp10* or *omp19* deletion on *Brucella abortus* outer membrane properties and virulence in mice. *Infect. Immun.* 70, 5540–6. doi:10.1128/IAI.70.10.5540-5546.2002.

Zúñiga-Ripa, A., Barbier, T., Lázaro-Antón, L., de Miguel, M. J., Conde-Álvarez, R., Muñoz, P. M., et al. (2018). The Fast-Growing *Brucella suis* Biovar 5 Depends on Phosphoenolpyruvate Carboxykinase and Pyruvate Phosphate Dikinase but Not on Fbp and GlpX Fructose-1,6-Bisphosphatases or Isocitrate Lyase for Full Virulence in Laboratory Models. *Front. Microbiol.* 9, 641. doi:10.3389/fmicb.2018.00641.
