# Supplementary material for: WadD, a New Brucella Lipopolysaccharide Core Glycosyltransferase Identified by Genomic Search and Phenotypic Characterization
Source: Front Microbiol. 2018 Sep 27;9:2293. doi: 10.3389/fmicb.2018.02293 (PMC6171495; doi:10.3389/fmicb.2018.02293)
Supplement: Supplementary file 7 [file Data_Sheet_7.PDF]

Table S2. Hypothetical glycosyltransferases identified in *B. abortus* and their orthologues in *Brucella spp*.

**Specie**	**ORF and presence**										
B. abortus	**BAB1_1620** (260 aa[1])	**BAB1_0326** (630 aa)	**BAB2_0133** (326 aa)	**BAB2_0135** (478 aa)	**BAB1_0953** (323 aa)	**BAB2_0693** (614 aa)	**BAB1_0607** (718 aa)	**BAB1_0114** (763 aa)	**BAB1_0932** (819 aa)	**BAB1_0417** (168 aa)	**BAB2_0105** (330 aa)
B. melitensis	✓[2]	✓	✓	✓[4]	✓	✓[5]	✓	✓[7]	✓	✓	✓
***B. suis* bv. 1**	✓	✓	✓	✓	✓	✓	✓	✓	✓	✓	✓
***B. suis* bv. 2**	✓	✓	✓	✓	✓	✗	✓	✓	✓	✓	✓
B. canis	✓	✓	✓	✓	✓	✓	✓	✓	✓	✗	✓
B. ovis	✓	✓[3]	✓	✓[4]	✓	✗	✓[6]	✓[7]	✓	✓	✓[8]
B. microti	✓	✓	✓	✓	✓	✓	✓	✓	✓	✓	✓
B. pinnipedialis	✓	✓	✓	✓	✓	✓	✓	✓	✓	✓	✓

[1] aa: aminoacids

✓ Ortholog present (when the symbol is alone, the ortholog is 100% identical to *B. abortus* sequence).

✗ Frameshift leading to a premature stop.

[2] 8 aminoacids longer (N-terminal) than *B. abortus*.

[3] 57 aminoacids shorter (N-terminal) than *B. abortus*.

[4] 1 aminoacid shorter (N-terminal) than *B. abortus*.

[5] 142 aminoacids shorter (N-terminal) than *B. abortus*.

[6] 6 aminoacids longer (N-terminal) than *B. abortus*.

[7] 33 aminoacids shorter (N-terminal) than *B. abortus*.

[8] 21 aminoacids longer (N-terminal) than *B. abortus*.
